# Supplementary material for: Shared regulatory networks link vein positioning and eyespot ring formation in butterflies
Source: Commun Biol. 2025 Dec 13;9:87. doi: 10.1038/s42003-025-09356-2 (PMC12820215; doi:10.1038/s42003-025-09356-2)
Supplement: Supplementary file 2 — Supplementary Information [file 42003_2025_9356_MOESM2_ESM.pdf]

Supplementary materials

Shared regulatory networks link vein positioning and eyespot ring formation in butterflies

Tirtha Das Banerjee [1, *] and Antónia Monteiro[1,2, *]

Affiliations
1 Department of Biological Sciences, National University of Singapore, Singapore - 117557.
2 Science Division, Yale-NUS College, Singapore - 138527.
* Authors for correspondence
Email: tirtha_banerjee@u.nus.edu, antonia.monterio@nus.edu.sg

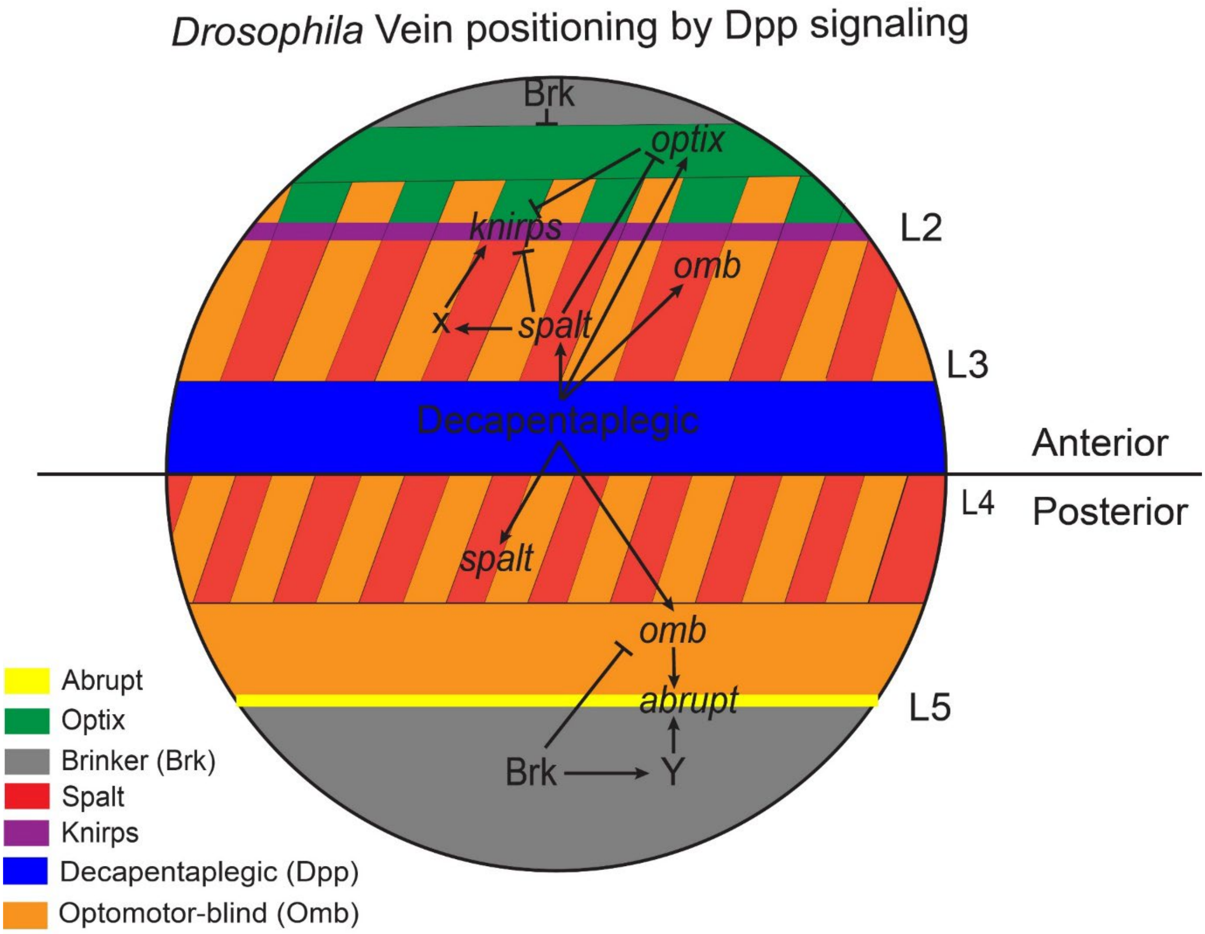

Supplementary Figure 1: Venation patterning in *Drosophila melanogaster* via Dpp signaling. High levels of Dpp activated at the AP boundary (between L3 and L4 veins) is capable of diffusing to the most anterior part of the larval wing disc where it activates key genes such as *Optix*, *omb* and *spalt* at different concentration thresholds, and is repressed by the transcription factor Brinker [1–4]. Spalt protein represses the expression of *Optix* in the anterior compartment [5]. Spalt also activates the transcription factor *knirps* via a hypothetical

protein X [6], while both Spalt and Optix repress *knirps* [5] which defines the fate of the L2 vein [5]. In the posterior compartment, Omb activates the transcription factor *abrupt* [7] while Brinker represses *omb* [8,9]. Brinker is also responsible for the activation of *abrupt* [7]. Abrupt protein is responsible for the formation of the L5 vein [7].

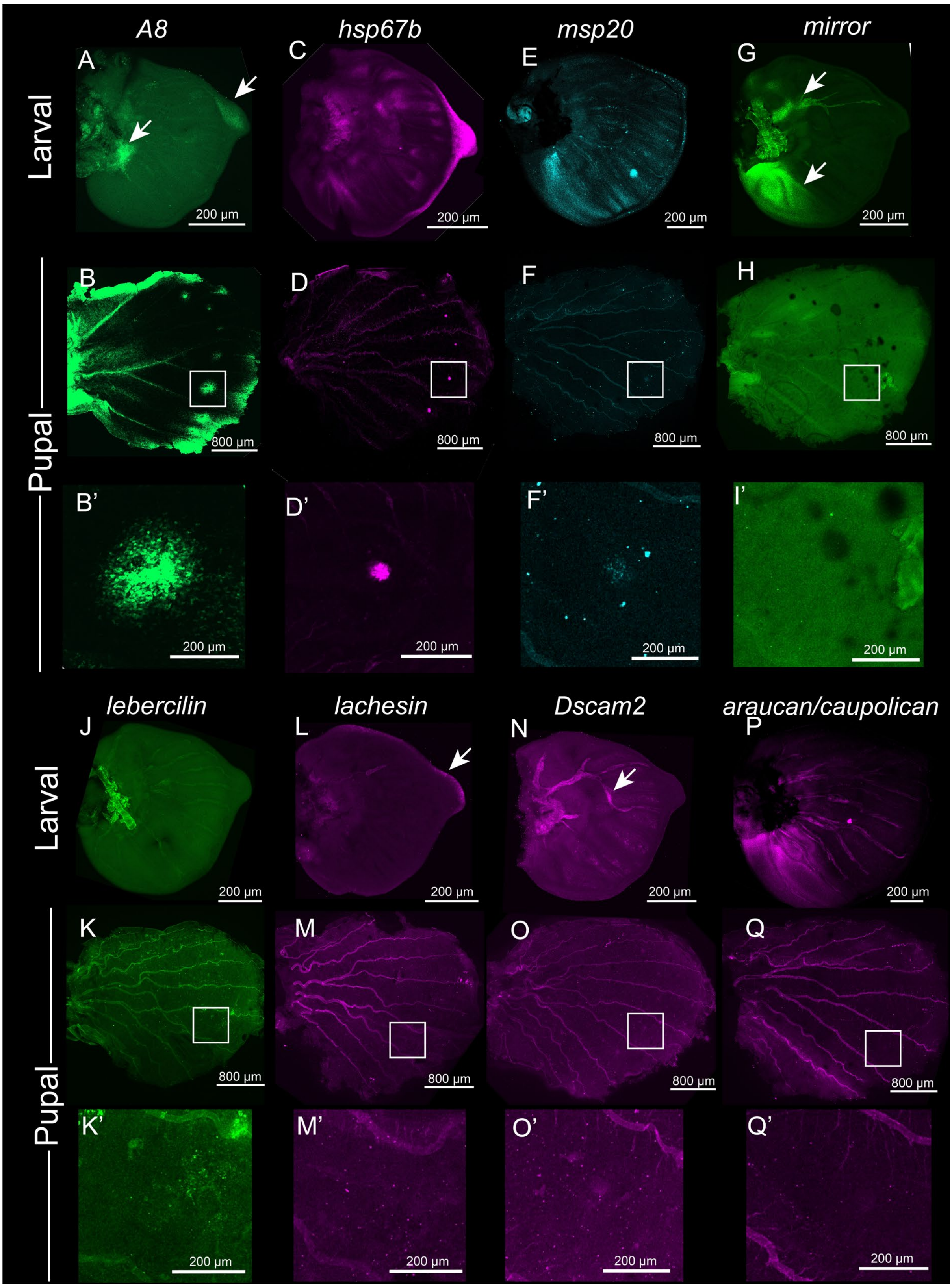

Supplementary Figure 2. Expression of *A8, hsp67b, msp20, mirror, lebercilin, lachesin, Dscam2,* and *araucan/caupolican* during larval and pupal wing development. (A) During the larval stage *A8* was expressed in two domains (white arrow). (**B**) During the pupal stage *A8* expression was observed strongly in the wing margin, in the eyespot center and the black scale cell domains. (**C**) *hsp67b* expression was observed in a complex domain during the

larval stage. (**D**) During the pupal stage, *hsp67b* expression was strongly observed in the center of the eyespots. (**E**) *msp20* expression was strongly observed in a single eyespot (Cu1) during the larval stage, along with stronger expression in the lower posterior compartment. (**F**) During the pupal stage, *msp20* expression continues in the Cu1 eyespot. (**G**) *mirror* expression was observed in the lower posterior compartment and in a proximal domain along the AP boundary. (**H**) During the pupal stage, no specific domain of *mirror* was observed. *lebercilin* didn't show any specific expression during the **(J)** larval stage and (**K**) pupal. **(L)** During the larval stage, *lachesin* was expressed in the anterior compartment along the wing margin (white arrow). (**M**) During the pupal stage, *lachesin* didn't show any specific expression domain. **(N)** During the larval stage, *Dscam3* was expressed in the discal spot. (**O**) During the pupal stage, *Dscam2* didn't showed any eyespot specific expression. **(P)** During the larval stage, *araucan/caupolican* was expressed strongly in the lower posterior compartment. (**Q**) During the pupal stage, slightly elevated levels of *araucan/caupolican* were observed in the lower posterior compartment with no eyespot specific expression.

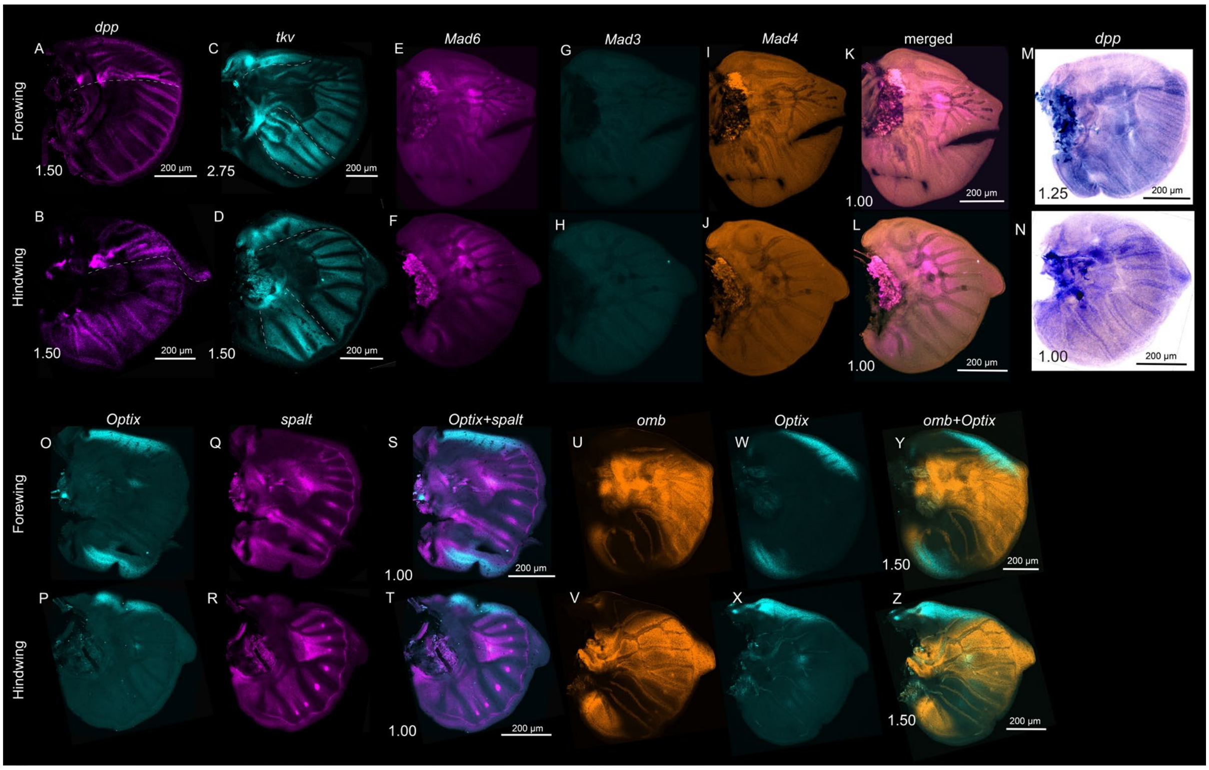

Supplementary Figure 3. Expression of *dpp, tkv, Mad6, Mad3, Mad4, Optix, spalt,* and *omb.* (A-Z) Expression of *dpp, tkv, Mad6, Mad3, Mad4, Optix*, *spalt*, and *omb* in the larval forewing and hindwing. No expression of *Mad3* and *Mad4* was observed.

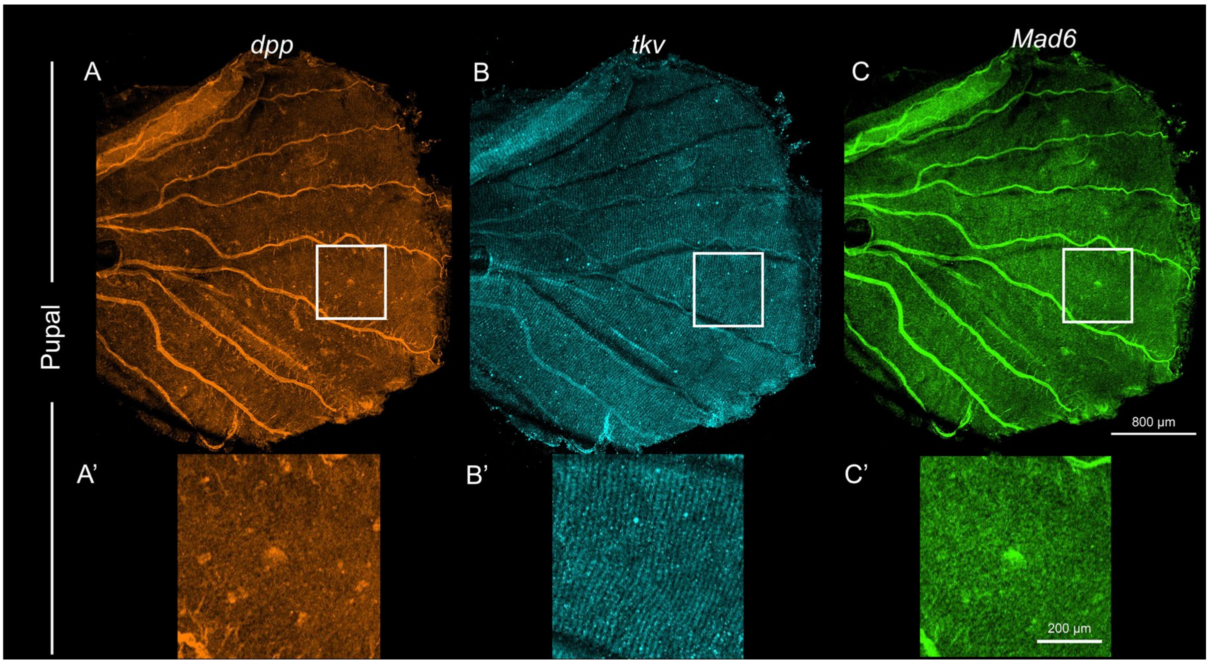

Supplementary Figure 4. Expression of *dpp, tkv*, and *Mad6* in a pupal wing of *B. anynana*. Expression of (**A**) *dpp* in the center of the eyespots, (**B**) *tkv* in the intervein cells, and (**C**) *Mad6* in the eyespot center and in the surrounding cells.

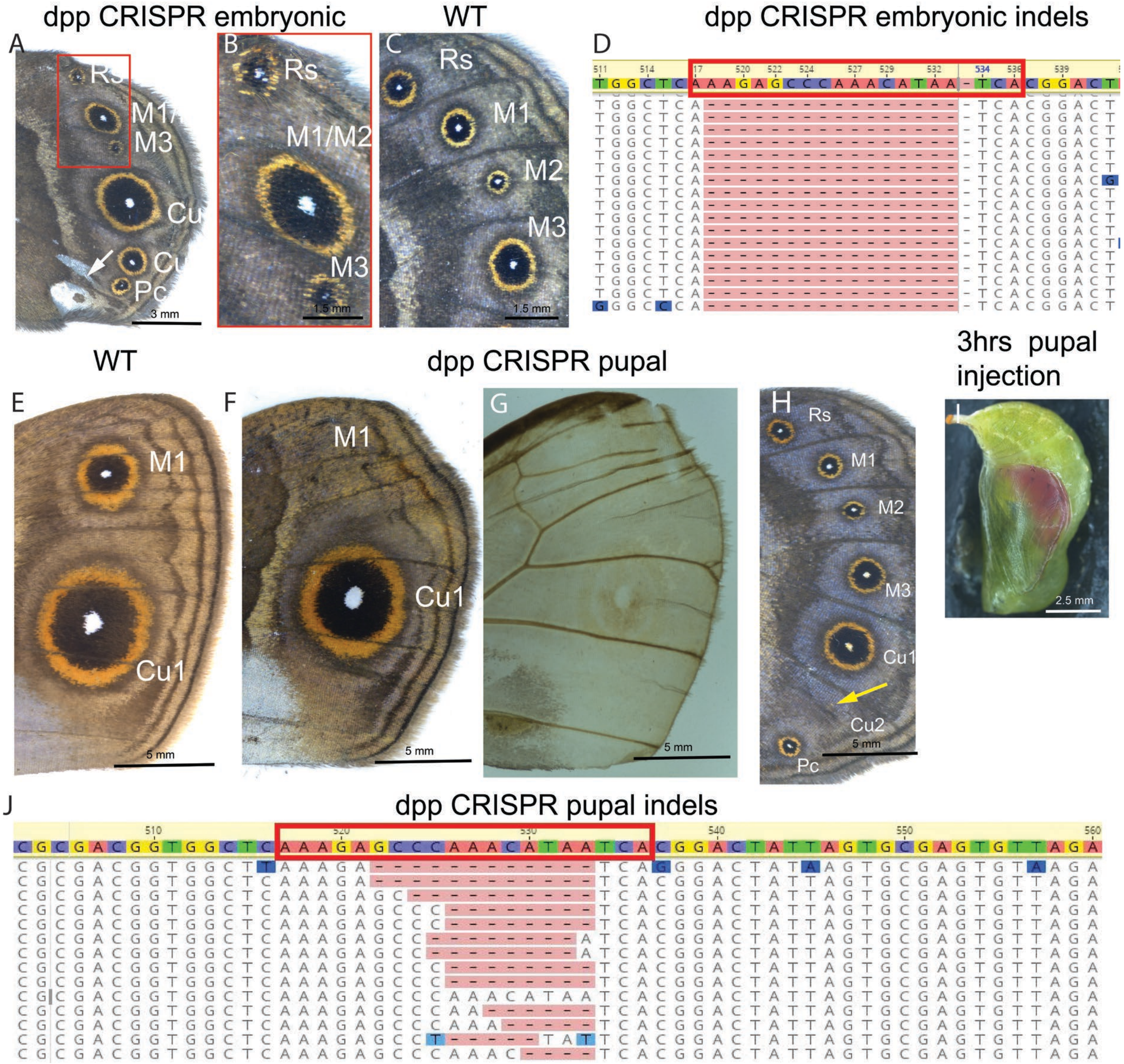

Supplementary Figure 5: Effect of *dpp* CRISPR on venation and eyespot development. (A and B) CRISPR-Cas9 injections of *dpp* during late embryonic development (6 hrs) resulted in the loss of either the M1 or M2 eyespot, increases in size of two eyespots M1 (or M2) and Cu1, and ectopic appearance of silver scales (white arrow) in a single individual (out of ~5000). **(C)** Adult WT hindwing. **(D)** Deletions (red box) at 3 bps 5' of the PAM sequence of the *dpp* guide RNA site (boxed area) obtained using DNA extracted from the wing shown in panels **A and B.** (**E**) WT adult forewing. (**F-H**) Pupal CRISPR-Cas9 injections using CRISPRMAX carrier resulted in a crispant showing loss of the anterior eyespot and disruptions in the M1 vein; and an individual with loss of Cu2 vein. (**I**) A 3 hr old pupa showing the injected medium (in red). (**J**) Indels at the site of *dpp* CRISPR from the wing in panel **F**. Embryonic injections resulted in high embryonic mortality. This is likely due to *dpp* being involved in patterning the early embryos. The red box indicate the CIRISPR target site.

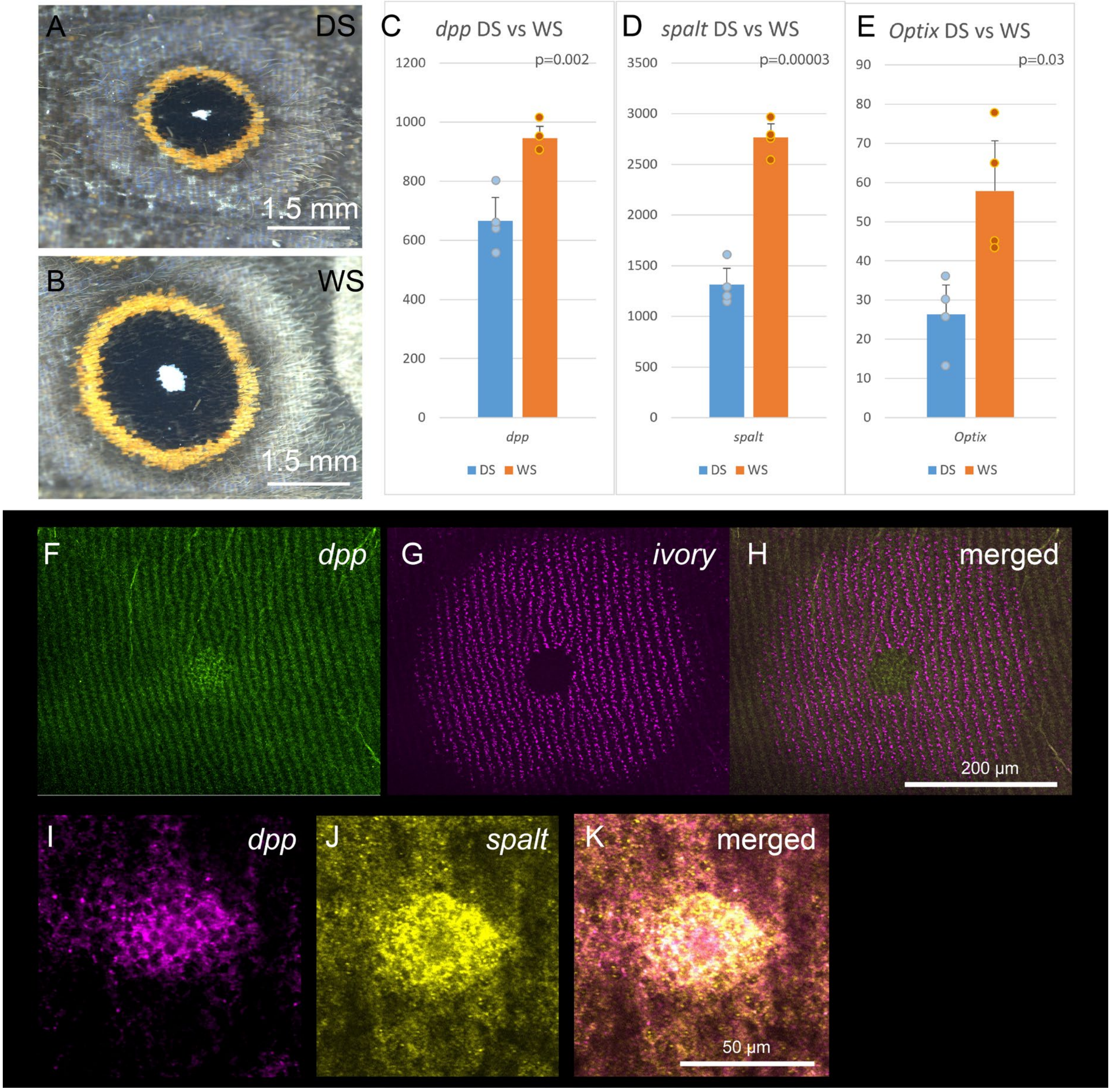

Supplementary Figure 6. Differential expression of *dpp, spalt,* and *Optix* in dry deason (DS) and wet season (WS) forms, and the expression of *dpp, ivory,* and *spalt* in 20-24 hrs pupal wings. WT (**A**) DS and (**B**) WS eyespots. Eyespots are smaller in DS season forms reared at a lower temperature. Differential expression of (**C**) *dpp*, (**D**) *spalt*, and (**E**) *Optix* in the DS and WS forms (data from [10]). Smaller domains of *spalt* and *Optix* are also observed in DS forms compared to WS forms [10]. For the RNAseq methodology for DS and WS eyespot data, please check [10]. Sample size for each type: 4 (Laser microdissection mediated RNAseq); Statistical test: ANOVA single factor; Error bars are standard deviation. (**F-K**) Expression of *dpp, spalt*, and *ivory* shows that the expression of *dpp* is restricted to the white center of the eyespots. *ivory* is only expressed in the black scales of the eyespots and is absent in the eyespot white centers [11].

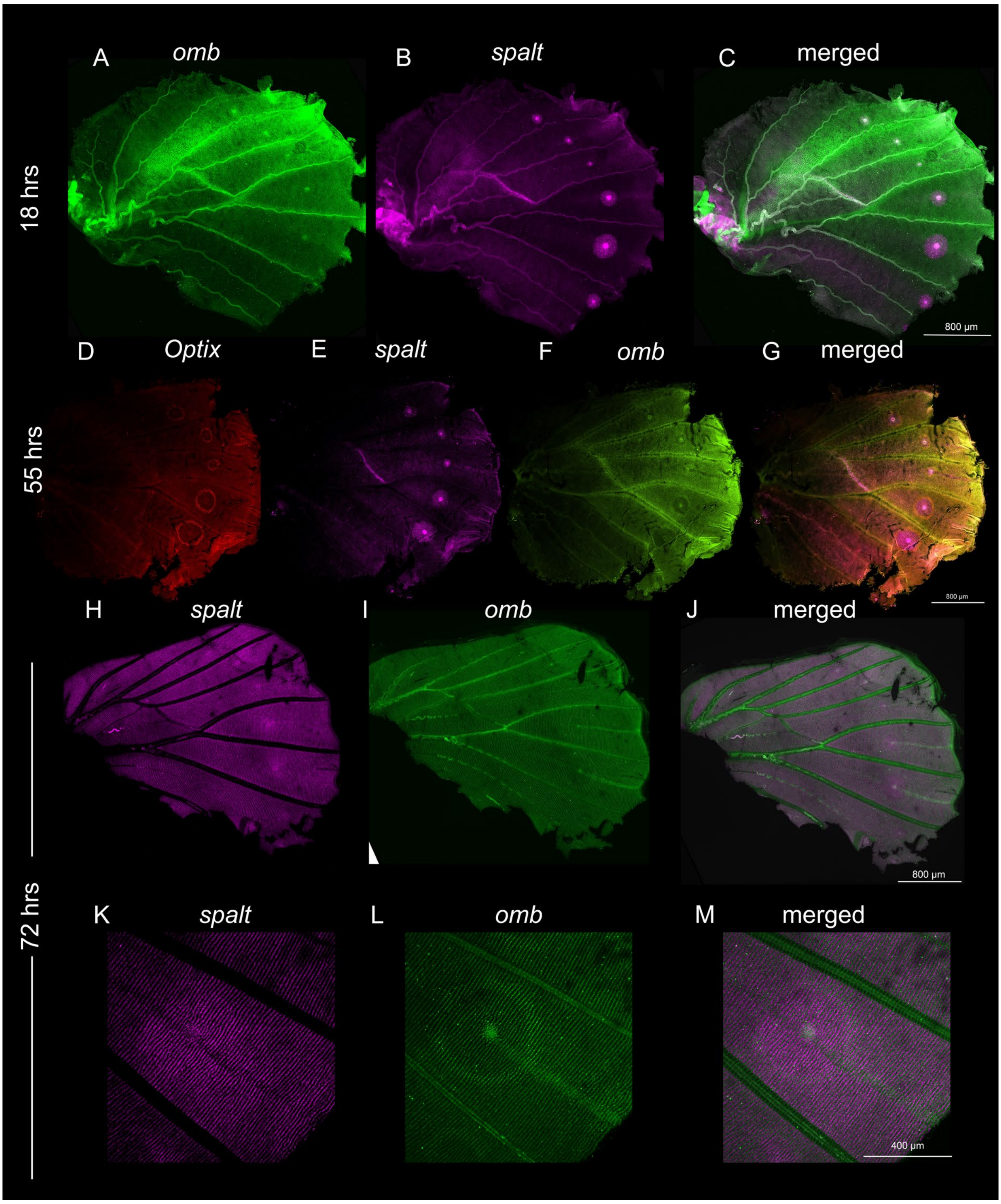

Supplementary Figure 7. Expression of *Optix, spalt*, and *omb* in 18-55 hrs pupal wings. (**A-C**) Expression of *spalt* and *Optix* in the 18 hrs pupal wings. (**D-G**) Expression of *Optix, spalt,* and *omb* in 55 hrs pupal wings. (**H-M**) Expression of *Optix* and *omb* in 72 hrs pupal wings. During the pupal wing development *omb* is expressed in the eyespot center, orange ring and along the conserved expression spanning the AP boundary, *spalt* is expressed in the eyespot center and the black disc cells, and *Optix* in the orange rings and along bands spanning the proximal-distal axis.

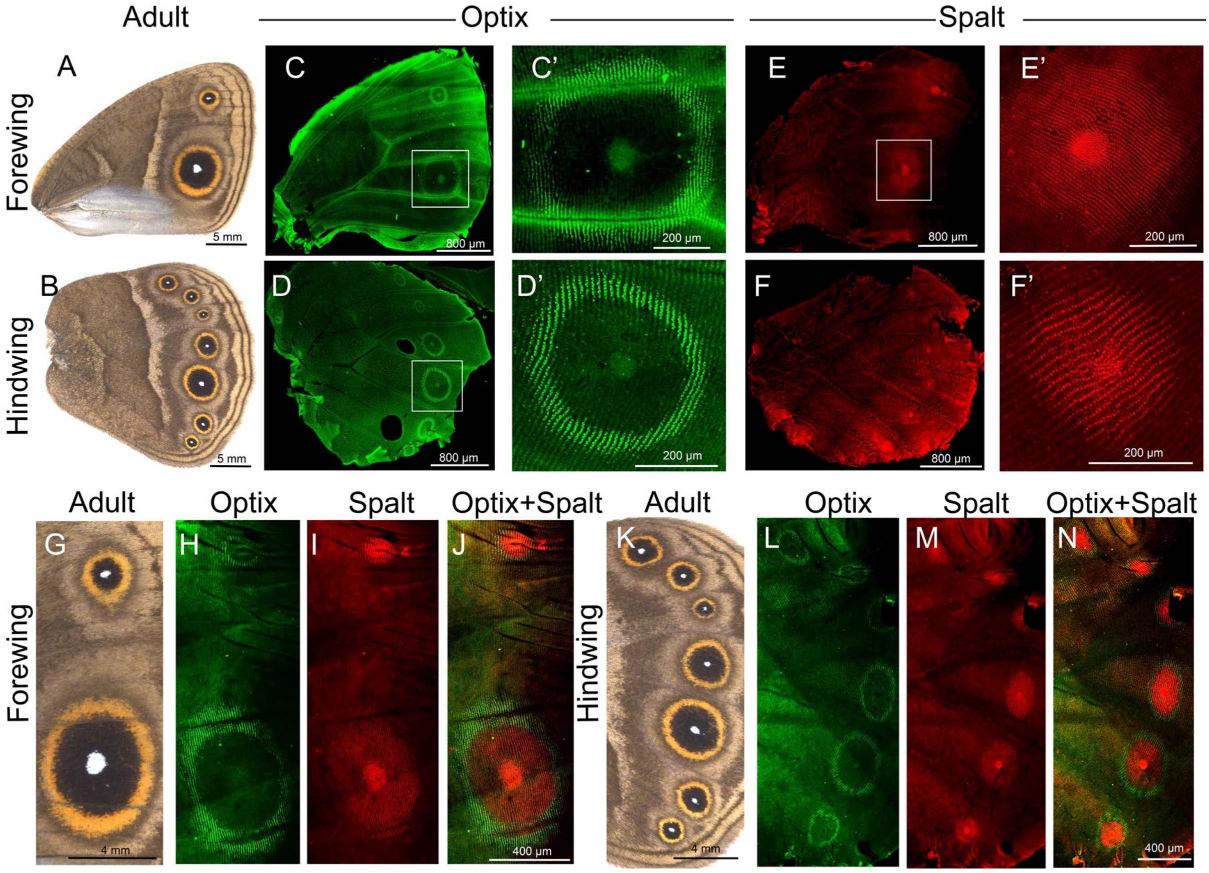

Supplementary Figure 8. ***B. anynana*** **adult wings and the localization of Optix and Spalt proteins in 16-44 hrs old pupal wings. (A)** WT forewing, **(B)** WT hindwing. **(C)** The presence of Optix proteins in the forewing and **(D)** in the hindwing. **(E and F)** Presence of Optix in the orange ring of Cu1 eyespots (boxed in C and D). (**G**) Presence of Spalt protein in the forewing and **(H)** in the hindwing. **(I and J)** Presence of Spalt protein in the black disc of Cu1 eyespots. (**G**) Adult forewing. (**H**) Optix localization in the forewing. (**I**) Spalt localization in the forewing. (**J**) Merged channels of Optix and Spalt. (**K**) Adult hindwing. (**L**) Optix localization in the hindwing. (**M**) Spalt localization in the hindwing. (**N**) Merged channels of Optix and Spalt.

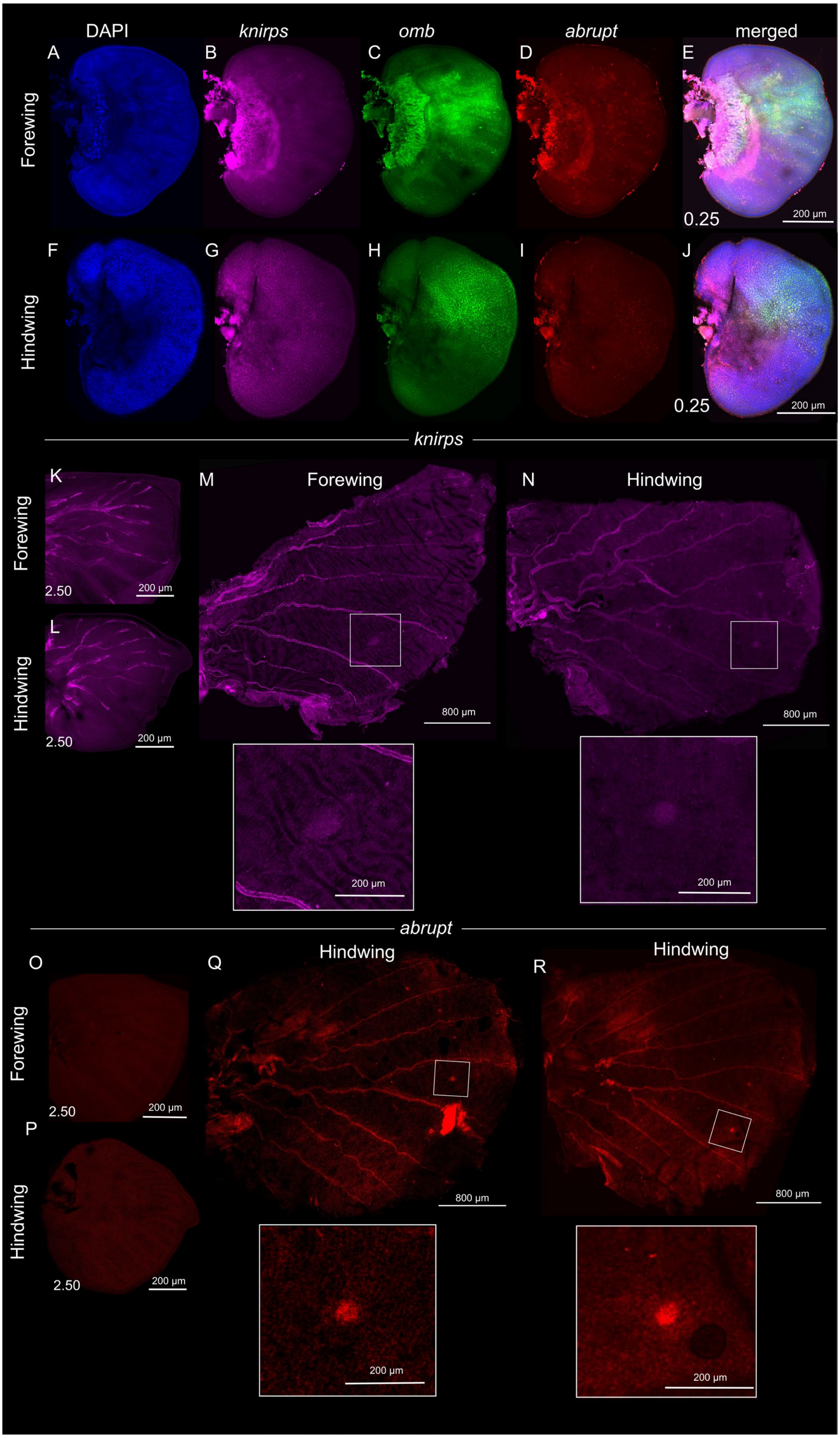
DAPI
knirps
omb
abrupt
merged
A
B
C
D
E
Forewing
0.25
200 µm
F
G
H
I
J
Hindwing
0.25
200 µm
knirps
K
Forewing
2.50
200 µm
L
Hindwing
2.50
200 µm
M
Forewing
800 µm
200 µm
N
Hindwing
800 µm
200 µm
abrupt
O
Forewing
2.50
200 µm
P
Hindwing
2.50
200 µm
Q
Hindwing
800 µm
200 µm
R
Hindwing
800 µm
200 µm

Supplementary Figure 9. Expression of *knirps*, *abrupt*, and *omb*. (A-J) Co-expression of DAPI, *knirps*, *omb*, and *abrupt* in early larval forewing and hindwing. *knirps* and *abrupt* didn't show any specific expression while *omb* was expressed in a broad AP domain and in the lower posterior domain. *knirps* didn't show any expression domain in the **(K, L)** late larval and **(M, N)** pupal wings. Expression of *abrupt* in the **(O, P)** late larval wing showing no specific expression domain and in the **(Q, R)** pupal wing showing expression in the eyespot centers.

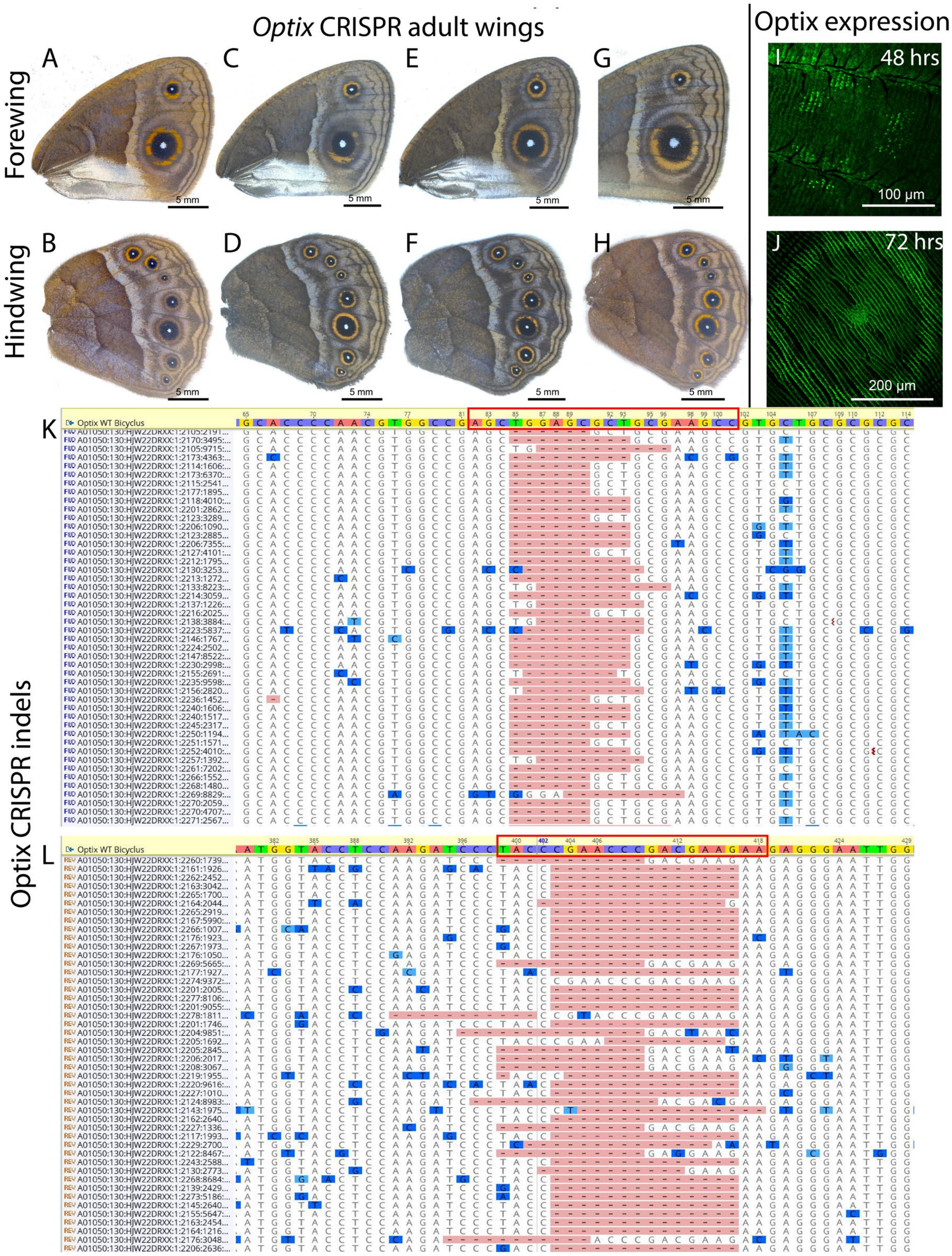

Supplementary Figure 10. Function of *Optix* in *Bicyclus anynana* butterflies and localization of Optix protein in pupal wings. (A-H) *Optix* crispants adult wings. *Optix*

knockouts result in the conversion of orange scales into brown scales in the eyespots. **(I and J)** Antibody staining of Optix proteins in an *Optix* crispant individual at 48 hrs and a WT 72 hrs old pupal wing. **(K and L)** Deletions at the two sites targeted for *Optix* CRISPR. The red boxes indicate the CIRISPR target site.

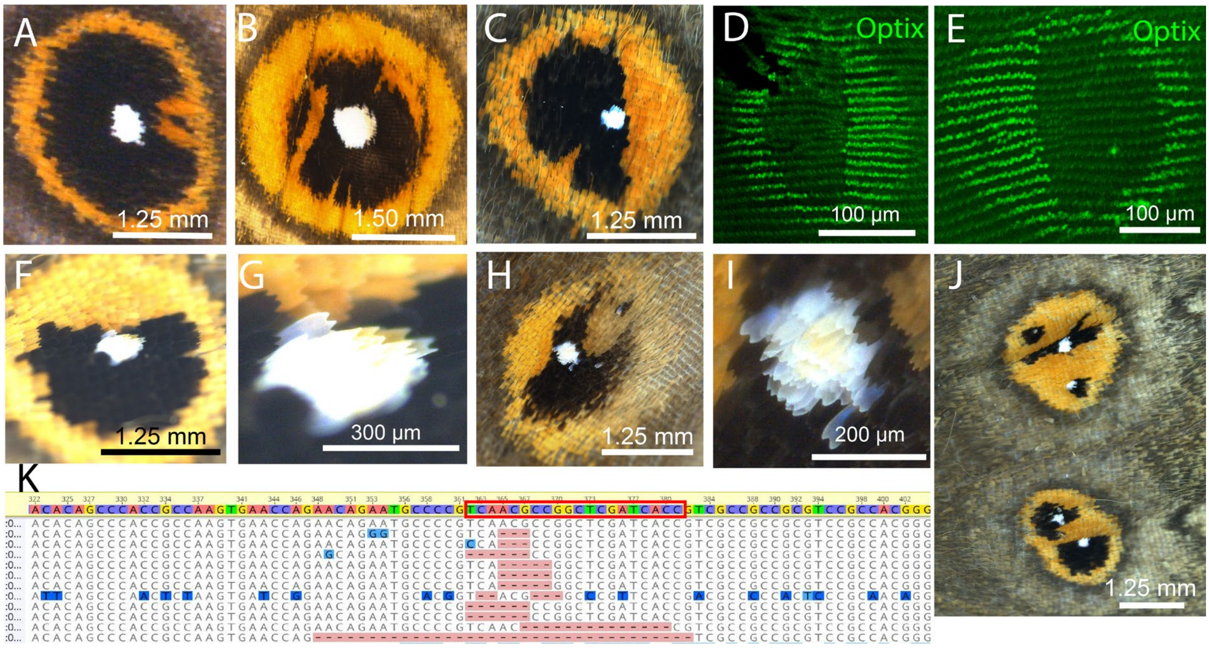

Supplementary Figure 11: Effect of *spalt* CRISPR on the eyespots. (**A-C**) Loss of Spalt results in the development of orange scales in the black scale region due to the presence of (**D and E**) Optix in that region. (**F-I**) Loss of *spalt* results in the development of yellow scales in the white center scale region of the eyespot. (**J**) Loss of *spalt* results in the split of eyespot foci with distinct domains of the white, black, and orange scales. (**K**) Deletions at the site of *spalt* CRISPR (red box). The red box indicate the CIRISPR target site.

Supplementary Table 1: Top differentially expressed genes in the anterior and posterior compartment of larval *B. anynana* wings.

**Gene_ID**	**Description**	**log2FoldChange**	**pvalue**	**padj**
LOC112054675	homeobox protein aristaless-like	3.073633	7.99E-24	8.79E-20
LOC112056678	homeobox protein aristaless	4.381925	2.21E-20	1.22E-16
LOC112043786	Down syndrome cell adhesion molecule-like protein Dscam2	9.676908	2.08E-12	7.62E-09
LOC112045125	hexamerin-like	-5.15048	4.66E-11	1.28E-07
LOC112057967	growth arrest-specific protein 1-like	2.251076	3.16E-10	6.96E-07
LOC112045741	homeobox protein caupolican-like	-2.84906	3.91E-09	7.17E-06
LOC112042874	tiggy-winkle hedgehog protein	-1.82419	4.87E-09	7.65E-06

LOC112047854	muscle segmentation homeobox-like	1.406152	1.58E-08	2.17E-05
LOC112046560	transcriptional activator cubitus interruptus isoform X1	2.869324	2.62E-08	3.20E-05
LOC112053006	homeobox protein invected-like isoform X2	-1.76609	4.74E-08	5.21E-05
LOC112048837	fork head domain transcription factor slp2-like	6.798241	2.60E-07	0.00026
LOC112057951	toll-like receptor 6	-1.84605	1.04E-06	0.000903
LOC112042983	lachesin-like	3.311257	1.07E-06	0.000903
LOC112053007	segmentation polarity homeobox protein engrailed-like	-1.60007	6.43E-06	0.005049
LOC112048760	neuroligin-2-like	2.85999	7.27E-06	0.005329
LOC112058231	uncharacterized protein LOC112058231	-8.11071	8.41E-06	0.005777
LOC112044740	dachshund homolog 2 isoform X2	2.011952	4.03E-05	0.026096
LOC128199477	#N/A	2.75725	5.41E-05	0.031285
LOC112053108	uncharacterized protein LOC112053108	1.331493	5.12E-05	0.031285
LOC112058471	carbonic anhydrase-related protein 10 isoform X1	2.456293	6.50E-05	0.035719
LOC112048640	T-box transcription factor TBX3 isoform X7	0.909847	7.43E-05	0.038906
LOC112054669	heat shock protein 67B1-like	1.189768	8.54E-05	0.041189
LOC112055169	ras-related protein Rab-32 isoform X5	-1.37732	8.61E-05	0.041189
LOC128198260	#N/A	0.903109	0.000101	0.046358
LOC112047794	rhomboid-related protein 3 isoform X1	-1.12963	0.000121	0.049466
LOC112047931	zinc finger protein ush isoform X1	-2.11728	0.00012	0.049466
LOC112045732	homeobox protein araucan-like isoform X1	-1.7553	0.000118	0.049466
LOC112051985	juvenile hormone epoxide hydrolase-like	7.410346	0.000143	0.055979
LOC112058319	muscle-specific protein 20-like	-1.19289	0.000153	0.058117
LOC112043273	uncharacterized protein LOC112043273	-7.15354	0.000255	0.089987
LOC112045364	uncharacterized protein LOC112045364	-0.99816	0.000262	0.089987
LOC112048596	putative inorganic phosphate cotransporter isoform X1	-1.10908	0.000261	0.089987
LOC112044525	homeobox protein SIX6-like	7.236613	0.000433	0.144285
LOC128199516	#N/A	-8.74784	0.000549	0.177527

LOC112058262	muscle, skeletal receptor tyrosine-protein kinase-like isoform X1	-1.24171	0.000646	0.203115
LOC112047337	toll-like receptor 7	0.78013	0.000702	0.214365
LOC112058222	lebercilin-like protein	7.045822	0.000741	0.220344
LOC112043001	uncharacterized protein LOC112043001	1.07768	0.000776	0.224482
LOC112048768	POU domain, class 6, transcription factor 2 isoform X1	-1.24136	0.000854	0.236967
LOC112054415	uncharacterized protein LOC112054415	0.83795	0.000862	0.236967
LOC112048478	protein mab-21	1.079178	0.000969	0.259859
LOC112050186	zinc finger protein 177-like	0.7199	0.001095	0.286586
LOC112051041	endocuticle structural glycoprotein ABD-5-like	2.864416	0.001126	0.287911
LOC112052161	protein patched isoform X1	0.902902	0.001162	0.290375
LOC112050109	probable chitinase 10	-1.04185	0.00126	0.308028
LOC112053786	uncharacterized protein LOC112053786	1.102876	0.001338	0.319755
LOC112055429	alaserpin-like isoform X1	0.69989	0.001534	0.358876
LOC112048344	cholinesterase 2-like	0.728368	0.00161	0.368844
LOC112043999	large neutral amino acids transporter small subunit 1	-0.5963	0.002085	0.467831

Supplementary Table 2: Top differentially expressed genes in the eyespot and control tissue of *B. anynana* pupal wings.

Gene_ID	Description	log2FoldChange	pvalue	padj
LOC112055331	protein obstructor-E-like	-4.938008557	5.16E-43	5.45E-39
LOC128199092	#N/A	-3.961160116	1.04E-39	5.48E-36
LOC112046720	serine/arginine repetitive matrix protein 1 isoform X1	-2.265975363	1.09E-32	3.83E-29
LOC128199053	#N/A	-4.121680054	1.38E-30	3.64E-27
LOC128199054	#N/A	-4.144273478	6.78E-29	1.43E-25
LOC112053683	G-patch domain and KOW motifs-containing protein, partial	-3.038345973	1.78E-28	3.14E-25
LOC128199095	#N/A	-4.765663963	1.63E-27	2.46E-24
LOC112050679	ras GTPase-activating protein-binding protein 2	2.030696914	7.08E-27	9.10E-24
LOC112053205	protein Peter pan	-2.555856714	7.76E-27	9.10E-24
LOC112043100	uncharacterized protein LOC112043100	-3.306811444	1.37E-26	1.45E-23
LOC112051683	uncharacterized protein LOC112051683	-2.781028756	2.57E-26	2.46E-23
LOC112054299	chromatin complexes subunit BAP18 isoform X2	-5.162649496	6.39E-26	5.62E-23

LOC128199083	#N/A	-4.188832582	1.17E-25	9.48E-23
LOC128199074	#N/A	-5.439863652	3.29E-25	2.48E-22
LOC112043561	ESF1 homolog	-2.309304506	3.19E-24	2.24E-21
LOC128199091	#N/A	-4.649886991	5.92E-24	3.91E-21
LOC112047762	MKI67 FHA domain-interacting nucleolar phosphoprotein-like	-2.158151544	1.45E-23	8.98E-21
LOC112048198	FACT complex subunit Ssrp1	-2.512173077	8.91E-23	5.22E-20
LOC128199080	#N/A	-4.523848077	9.43E-23	5.24E-20
LOC128199096	#N/A	-4.42987424	2.48E-22	1.31E-19
LOC112053191	elongin-B isoform X2	1.755529117	4.52E-22	2.27E-19
LOC112048594	armadillo segment polarity protein isoform X2	1.916341092	1.11E-21	5.33E-19
LOC112056205	U3 small nucleolar ribonucleoprotein protein MPP10	-2.110440974	3.38E-21	1.55E-18
LOC112045285	ethanolaminephosphotransferase 1-like	2.150614042	6.41E-21	2.82E-18
LOC112054861	RNA-binding protein 28	-2.286205593	7.02E-21	2.97E-18
LOC128199067	#N/A	-3.583703971	1.44E-20	5.83E-18
LOC128199079	#N/A	-4.411317293	1.91E-20	7.46E-18
LOC112046874	zinc finger protein on ecdysone puffs-like isoform X1	-1.971125298	2.98E-20	1.12E-17
LOC112050585	protein PELPK1-like	-4.155464865	1.45E-19	5.28E-17
LOC112049406	pre-mRNA-splicing factor Slu7	-2.192332327	1.52E-19	5.35E-17
LOC112056909	probable ATP-dependent RNA helicase Dbp45A isoform X2	-2.252771749	1.92E-19	6.55E-17
LOC112045966	pupal cuticle protein PCP52-like	-2.72140226	2.08E-19	6.87E-17
LOC128199087	#N/A	-4.381284748	3.21E-19	1.03E-16
LOC128199101	#N/A	-4.898938121	4.58E-19	1.42E-16
LOC112044446	polycomb protein Sfmbt-like	2.247138379	6.07E-19	1.83E-16
LOC128199088	#N/A	-4.071406839	8.35E-19	2.45E-16
LOC112046711	nucleolar protein 8	-1.850564311	9.17E-19	2.62E-16
LOC112055320	tyrosine-protein phosphatase non-receptor type 9	2.834548157	2.27E-18	6.31E-16
LOC112044989	pupal cuticle protein G1A-like	-5.652936924	2.36E-18	6.38E-16
LOC112044964	uncharacterized protein LOC112044964	-4.887939958	2.42E-18	6.39E-16
LOC112058154	calcium homeostasis endoplasmic reticulum protein isoform X1	-1.958560549	2.84E-18	7.30E-16
LOC112052258	protein SDA1 homolog	-2.10131782	3.14E-18	7.88E-16
LOC112045120	uncharacterized protein LOC112045120	1.889710604	7.80E-18	1.91E-15
LOC112051814	protein TIS11	1.745206078	9.87E-18	2.37E-15
LOC112048699	DNA-(apurinic or apyrimidinic site) lyase-like	-1.905162827	1.20E-17	2.81E-15
LOC112048503	ubiquitin-conjugating enzyme E2-24 kDa isoform X1	2.592535368	1.69E-17	3.88E-15
LOC112046604	surfeit locus protein 6 homolog	-1.606657809	3.10E-17	6.97E-15
LOC128199052	#N/A	-4.901297572	3.52E-17	7.74E-15
LOC112046338	failed axon connections	1.536392399	4.11E-17	8.84E-15
LOC112047699	uncharacterized protein LOC112047699	-1.849746015	4.41E-17	9.13E-15
LOC112049355	pre-mRNA-splicing factor CWC22 homolog	-2.576363811	4.40E-17	9.13E-15

LOC112047981	uncharacterized protein C14orf119 homolog	1.793680976	5.61E-17	1.14E-14
LOC112052912	pre-rRNA processing protein FTSJ3	-2.073395186	6.26E-17	1.25E-14
LOC112055040	peptidyl-prolyl cis-trans isomerase D	1.708616076	8.66E-17	1.69E-14
LOC112057782	homeotic protein ultrabithorax	1.919667063	1.10E-16	2.12E-14
LOC112046508	uncharacterized protein LOC112046508	1.48893366	1.21E-16	2.27E-14
LOC112050572	sister chromatid cohesion protein PDS5 homolog B-like	2.07540653	1.66E-16	3.07E-14
LOC112045283	partitioning defective 6 homolog beta	1.992281153	1.71E-16	3.11E-14
LOC112053604	arginine/serine-rich protein PNISR	-1.94743651	2.81E-16	5.03E-14

Supplementary Table 3: Primer and CRISPR guide table

Sl. No.	Name	Sequence
1.	dpp_insitu_F	GTTCTTCAACGTAAGCGGCG
2.	dpp_insitu_R	CCACAGCCTACCACCATCAT
3.	dpp_sequencing_F	GCCTGTTCTTCAACGTAAGC
4.	dpp_sequencing_R	CTCCGTGTACAGCATGAGC
5.	dpp_sequencing300_F	ACCGGCAGACAGAGACTG
6.	dpp_sequencing300_R	CCACTCCTCCTCCTCGTC
5.	optix_sequencing_F	AGACGCTGGAGGAGAGC
6.	optix_Sequencing_R	CGCTCGGTCTCTTTGC
7.	spalt_sequencing_F	GCATCGACAAGATGCTGAAA
8.	spalt_Sequencing_R	TTCATTTAGGGACGGTGGAG
9.	optix_CRISPR_1	GAAATTAATACGACTCACTATAGGGGCTTCGCAG CGCTCCAGCTGTTTTAGAGCTAGAAATAGC
10.	optix_CRISPR_2	GAAATTAATACGACTCACTATAGGTTCTTCGTCGG GTTCGGGTAGTTTTAGAGCTAGAAATAGC
11.	spalt_CRISPR_1	GAAATTAATACGACTCACTATAGGTGATCGAGCC GGCGTTGAGTTTTAGAGCTAGAAATAGC
12.	CRISPR_reverse	AAAAGCACCGACTCGGTGCCACTTTTT CAAGTTGATAACGGACTAGCCTTATTT TAACTTGCTATTTCTAGCTCTAAAAC

Note: Synthetic guide was ordered for dpp CRISPR.

Supplementary Table 4. Immunofluorescence Buffers

Buffers	Chemicals	Amount
Fix buffer (30 ml)	0.1M PIPES pH 6.9 (500 mM)	6 ml
	1 mM EGTA pH 6.9 (500mM)	60 µl
	1% Triton x-100 (20 %)	1.5 ml

	2 mM $MgSO_4$ (1M)	60 µl
	37% Formaldehyde	55 µl per 500 µl of buffer
	dH_2O	22.4 ml
Block buffer (40 ml)	50 mM Tris pH 6.8 (1 M)	2 ml
	150 mM NaCl (5 M)	1.2 ml
	0.5% IGEPAL (NP40) (20%)	1 ml
	5 mg/ml BSA	0.2 gr
	H2O	35.8 ml
Wash buffer (200 ml)	50mM Tris pH 6.8 (1 M)	10 ml
	150 mM NaCl (5 M)	6 ml
	0.5% IGEPAL (20 %)	5 ml
	1 mg/ml BSA	0.2 gr
	dH_2O	179 ml
Mounting media	Tris-HCl (pH 9.2)	20 mM
	N-propyl gallate	0.5%
	Glycerol	60%

Supplementary Table 5. Enzyme based *in-situ* hybridization Buffers.

Buffers	Chemicals	Amount
10X PBS (500 ml)<br>* Sterilize by autoclaving.	K_2HPO_4	5.34 g
	KH_2PO_4	2.64 g
	NaCl	40.9 g
	DEPC treated H_2O	To 500 ml
1X PBST (50 ml)	1X PBS	50 ml
	Tween® 20	50 µl
20X SSC (1000 ml)<br>*Adjust the pH to 7.0 with 1M HCl and sterilize by autoclaving.	NaCl	175.3 g
	Trisodium citrate	88.2 g
	DEPC treated H_2O	Till 1000 ml
	Formamide	20 ml

Pre-hybridization buffer (40 ml)	20X SSC	10 ml
	DEPC treated water	10 ml
	TWEEN20	40 µl
Hybridization buffer (40 ml)	Formamide	20 ml
	20X SSC	10 ml
	DEPC treated water	10 ml
	TWEEN20	40 µl
	Spaltmon sperm	40 µl
	Glycine (100mg/ml)	40 µl
Block buffer (50 ml)	1X PBS	50 ml
	TWEEN20	50 µl
	BSA	0.1 gm
Alkaline phosphatase buffer (20 ml)	Tris-HCl (pH 8.0)	2 ml
	NaCl (5M)	400 µl
	$MgCl_2$ (200mM)	250 µl
	DEPC treated water	Till 20 ml
	TWEEN20	20 µl

Supplementary Note 1: Sequences of genes and oligos

The section contains the gene and probe sequences for in-situs and CRISPR.

Sequence of *decapentaplegic* used for enzyme based *in-situ* hybridization.

GTTCTTCAACGTAAGCGGCGTACCGGCCGACGAGGTGGCGCGCGGCGCCGACCTCTCGTT
CCAACGAGCCGTCGGCACCACCGGCAGACAGAGACTGTTGTTGTACGACGTGGTGCGCC
CTGGCCGCCGCGGCCACTCCGAGCCGATCCTGCGGCTGCTGGACTCCGTTCCGCTCCGGC
CCGGGGAGGGAATCGTCAACGCCGACGCTCTGGGAGCGGCGCGACGGTGGCTCAAAGA
GCCCAAACATAATCACGGACTATTAGTGCGAGTGTTAGAAGAAGACGCCGCGAGTGCGA
GCAGGGACGCGAAGTTCCCGCACGTGCGCGTGCGCAGACGCGTCACGGACGAGGAGGA
GGAGTGGCGGACGGCGCAGCCGCTGCTCATGCTGTACACGGAGGACGAGCGCGCGCGCG
CGTCGCGGGAGACGAGCGAGCGGCTGACGCGCAGCAAGCGCGCGGCGCAGCGGCGGGG
GCACCGCGCGCACCACCGCCGCAAGGAGGCGCGCGAGATCTGCCAGCGCCGCCCGCTGT
TCGTCGACTTCGCGGACGTGGGCTGGAGCGACTGGATCGTGGCCCCGCACGGCTACGAC
GCGTACTACTGCCAGGGCGACTGCCCCTTCCCGCTGCCGGACCACCTCAACGGCACGAAC
CACGCGATAGTGCAGACTCTGGTCAACTCAGTGAACCCCGCGACGGTGCCCAAAGCGTG

CTGCGTGCCGACGCAACTCTCATCTATATCTATGTTATATATGGACGAAGTGAACAATGT
GGTGCTTAAAAACTATCAGGACATGATGGTGGTAGGCTGTGG

Region of *dpp* used for CRISPR-Cas9 (Highlighted in red)

ATGCGTGGGGCGTGCGCGTGCGCGGTGGTGTGCGCGTTGGTGGCGCTGTGCGCG
GCGCGGCTGGACGAGTCCGCGCGCGCCGCCGCCGAGAAGCAGCTGCTGGCACTG
CTGGGCCTGCCGCGCCGGCCGCCGCCGCGCGCCCGCCCGCCGCCCGTGCCGCGC
GCGCTGCGCGTGCTGTACGACTCGCGCGCGCTGCCCGCCGCCGCCGCCAACACG
GCGCGCTCCTTCCACCACACGCCCACGCCGCTCGACGAGCGCTTCCCCGGCGACC
ACCGCTTCCGCCTGTTCTTCAACGTAAGCGGCGTACCGGCCGACGAGGTGGCGCG
CGGCGCCGACCTCTCGTTCCAACGAGCCGTCGGCACCACCGGCAGACAGAGACT
GTTGTTGTACGACGTGGTGCGCCCTGGCCGCCGCGGCCACTCCGAGCCGATCCTG
CGGCTGCTGGACTCCGTTCCGCTCCGGCCCGGGGAGGGAATCGTCAACGCCGAC
GCTCTGGGAGCGGCGCGACGGTGGCTCAAAGAGCCCAAACATAATCACGGACTA
TTAGTGCGAGTGTTAGAAGAAGACGCCGCGAGTGCGAGCAGGGACGCGAAGTTC
CCGCACGTGCGCGTGCGCAGACGCGTCACGGACGAGGAGGAGGAGTGGCGGAC
GGCGCAGCCGCTGCTCATGCTGTACACGGAGGACGAGCGCGCGCGCGCGTCGCG
GGAGACGAGCGAGCGGCTGACGCGCAGCAAGCGCGCGGCGCAGCGGCGGGGGC
ACCGCGCGCACCACCGCCGCAAGGAGGCGCGCGAGATCTGCCAGCGCCGCCCGC
TGTTCGTCGACTTCGCGGACGTGGGCTGGAGCGACTGGATCGTGGCCCCGCACG
GCTACGACGCGTACTACTGCCAGGGCGACTGCCCCTTCCCGCTGCCGGACCACCT
CAACGGCACGAACCACGCGATAGTGCAGACTCTGGTCAACTCAGTGAACCCCGC
GACGGTGCCCAAAGCGTGCTGCGTGCCGACGCAACTCTCATCTATATCTATGTTA
TATATGGACGAAGTGAACAATGTGGTGCTTAAAAACTATCAGGACATGATGGTG
GTAGGCTGTGGCTGCCGATGA

Region of *Optix* used for CRISPR-Cas9 (Highlighted in red)

ATGCGCGGCTCCTGGGACGAGTCCACGACGGCGGCGCTGCACGCGCGCATCCTGGAGGC
GCACCGCGGGTCCGCCGCGCCCGACCGCGCCGAGCCCGCGTGCGAGCCTCCGCCGCTGA
CGCTGGGCGCGCTGGAGCTGGCGGCGCCCACGCCGCTGCTGCCGCTGCCCACGCTGAGC
TTCAGCGCCGCGCAGGTGGCCACCGTGTGCGAGACGCTGGAGGAGAGCGGCGACGTGGA
GCGCCTGGCGCGCTTCTTGTGGTCGCTGCCCGTGGCGCACCCCAACGTGGCCGAGCTGGA
GCGCTGCGAAGCCGTGCTGCGCGCGCGCGCCGTCGTCGCCTTCCACGCCGGCCGCCACCG
CGAGCTGTACGCCATCCTCGAGCGCCACCGCTTCCAGCGCTCCAGCCACGCCAAGCTGCA
AGCGCTGTGGCTGGAGGCGCACTACCAGGAGGCTGAGCGCCTGCGCGGCCGTCCGCTGG
GCCCCGTCGACAAGTACCGCGTGCGGAAGAAGTTCCCGCTCCCGAGGACGATCTGGGAC
GGCGAGCAGAAGACGCACTGTTTCAAGGAGCGGACGCGATCTCTACTCCGAGAATGGTA
CCTCCAAGATCCCTACCCGAACCCGACGAAGAAGAGGGAATTGGCGGCGGCGACGGGTC
TGACGCCGACGCAAGTCGGCAACTGGTTCAAAAACCGACGGCAAAGAGACCGAGCGGC
CGCCGCCAAGAACCGCTCCGCCGTGCTGGGCAGAGGATAA

Region of *spalt* targeted by CRISPR-Cas9 (location of guide RNA highlighted in red)

GCATCGACAAGATGCTGAAAATAATAATAGTCTCGAAGACGGCGAGGCCGAAATACCTG
AAGCCGACATGCCCCCCGTGGGTCTGCCGTTCCCTTTGGCAGGACACGTTACTCTTGAGG
CTCTACAAAATACGAGAGTAGCGGTCGCCCAATTCGCTGCAACAGCGATGGCAAATAAT
GCGAATAACGAAGCTGCTATACAAGAATTACAAGTGTTACACAACACTCTATACACTTTA
CAGTCACAACAAGTATTTCAACTTCAGTTAATACGTCAGCTTCAGAATCAGTTATCTCTA

ACTCGACGGAAAGAAGACGATCCACACAGCCCACCGCCAAGTGAACCAGAACAGAATG
CCCCGTCAACGCCGGCTCGATCACCGTCGCCGCCGCGTCCGCCACGGGAGCCGTCGCCTG
TTATACCCTCTCCTCCTACTAGCCAAAGTTTGCCGTCGACTCACACACATCACACACCCA
AAACTGAACAGATATCTATCCCTAAGATTCCAACTTCCTCACCATCTTTAATGACCCACC
CACTTTATAGTTCAATTTCTTCGTCATTAGCATCTTCCATCATAACAAACAATGATCCTCC
ACCGTCCCTAAATGAA

Sequence of the genes and the oligos used for HCR3.0

decapentaplegic (dpp)

>decapentaplegic_B1_XM_052883655.1

GTTCTTCAACGTAAGCGGCGTACCGGCCGACGAGGTGGCGCGCGGCGCCGACCTCTCGTTCCAAC
GAGCCGTCGGCACCACCGGCAGACAGAGACTGTTGTTGTACGACGTGGTGCGCCCTGGCCGCCGC
GGCCACTCCGAGCCGATCCTGCGGCTGCTGGACTCCGTTCCGCTCCGGCCCGGGGAGGGAATCGTC
AACGCCGACGCTCTGGGAGCGGCGCGACGGTGGCTCAAAGAGCCCAAACATAATCACGGACTATT
AGTGCGAGTGTTAGAAGAAGACGCCGCGAGTGCGAGCAGGGACGCGAAGTTCCCGCACGTGCGC
GTGCGCAGACGCGTCACGGACGAGGAGGAGGAGTGGCGGACGGCGCAGCCGCTGCTCATGCTGTA
CACGGAGGACGAGCGCGCGCGCGCGTCGCGGGAGACGAGCGAGCGGCTGACGCGCAGCAAGCGC
GCGGCGCAGCGGCGGGGGCACCGCGCGCACCACCGCCGCAAGGAGGCGCGCGAGATCTGCCAGC
GCCGCCCGCTGTTCGTCGACTTCGCGGACGTGGGCTGGAGCGACTGGATCGTGGCCCCGCACGGCT
ACGACGCGTACTACTGCCAGGGCGACTGCCCCTTCCCGCTGCCGGACCACCTCAACGGCACGAAC
CACGCGATAGTGCAGACTCTGGTCAACTCAGTGAACCCCGCGACGGTGCCCAAAGCGTGCTGCGT
GCCGACGCAACTCTCATCTATATCTATGTTATATATGGACGAAGTGAACAATGTGGTGCTTAAAAA
CTATCAGGACATGATGGTGGTAGGCTGTGG

Dpp1_HCR_P1B1: gAggAgggCAgCAAACggAAACCTCGTCGGCCGGTACGCCGCTTA

Dpp1_HCR_P2B1: TGGAACGAGAGGTCGGCGCCGCGCGTAgAAgAgTCTTCCTTTACg

Dpp2_HCR_P1B1: gAggAgggCAgCAAACggAACAGTCTCTGTCTGCCGGTGGTGCCG

Dpp2_HCR_P2B1: GCCAGGGCGCACCACGTCGTACAACTAgAAgAgTCTTCCTTTACg

Dpp3_HCR_P1B1: gAggAgggCAgCAAACggAACAGCCGCAGGATCGGCTCGGAGTGG

Dpp3_HCR_P2B1: CCCGGGCCGGAGCGGAACGGAGTCCTAgAAgAgTCTTCCTTTACg

Dpp4_HCR_P1B1: gAggAgggCAgCAAACggAAGCCGCTCCCAGAGCGTCGGCGTTGA

Dpp4_HCR_P2B1: TGTTTGGGCTCTTTGAGCCACCGTCTAgAAgAgTCTTCCTTTACg

Dpp5_HCR_P1B1: gAggAgggCAgCAAACggAACGCCACTCCTCCTCCTCGTCCGTGA

Dpp5_HCR_P2B1: TACAGCATGAGCAGCGGCTGCGCCGTAgAAgAgTCTTCCTTTACg

Dpp6_HCR_P1B1: gAggAgggCAgCAAACggAATCTCCCGCGACGCGCGCGCGCGCTC

Dpp6_HCR_P2B1: GCTTGCTGCGCGTCAGCCGCTCGCTTAgAAgAgTCTTCCTTTACg

Dpp7_HCR_P1B1: gAggAgggCAgCAAACggAAGCCCACGTCCGCGAAGTCGACGAAC

Dpp7_HCR_P2B1: GTGCGGGGCCACGATCCAGTCGCTCTAgAAgAgTCTTCCTTTACg

Dpp8_HCR_P1B1: gAggAgggCAgCAAACggAAGGGGCAGTCGCCCTGGCAGTAGTAC

Dpp8_HCR_P2B1: GCCGTTGAGGTGGTCCGGCAGCGGGTAgAAgAgTCTTCCTTTACg

Dpp9_HCR_P1B1: gAggAgggCAgCAAACggAACACTGAGTTGACCAGAGTCTGCACT

Dpp9_HCR_P2B1: GCACGCTTTGGGCACCGTCGCGGGGTAgAAgAgTCTTCCTTTACg

Dpp10_HCR_P1B1: gAggAgggCAgCAAACggAAATATAACATAGATATAGATGAGAGT

Dpp10_HCR_P2B1: AAGCACCACATTGTTCACTTCGTCCTAgAAgAgTCTTCCTTTACg

optomotor-blind (omb)

>optomotor-blind_B1_XM_052882932.1

ATGCATCATCTCGAGAATTTCTCAATAAGCCGTGGTGCCTGTGGGGCCGACGCGGGCGCCGCCCCG
CGGCGAGTCGGTGGCGCCTCTATCGATTATTCGGGCCTCTCGGTCGCGCGTAGCGCCGCGTCCCGC
TCGCGCCGCGCGGGTGGCGCCCGCCGCGTCATGCGCGACCACCGCGCGACTGGCGCGACCTCGCG
CGAGCCGCTCTCGGGCTATATCATTAATCTATTACTCCGGGAAGGTGGGCTCGTACGTAAGGAGCT
CATCTATAGCCAAAGTCAACGGATGTTGTCAAAGTTGCTGTGGCCAAACCCAGTGCCTAGTCCGCC
CAGAGACCCCCACTCAGATCATTTGACGATCCTGAACTCGATGCACAAGTACCAGCCACGGTTCCA
CCTGGTGCGAGCCAACGACATCCTCAAGCTGCCCTACTCCACCTTCCGCACCTACGTATTCAAGGA
GACCGAGTTCATCGCCGTCACCGCCTACCAGAACGAGAAGATAACGCAGCTGAAAATCGACAACA
ACCCCTTCGCGAAAGGCTTCCGAGACACGGGGGCGGGGAAGCGGGAGAAGAATCTGTCCGTGTAC
CGCAGGCAGGCGCTGCTGACGGCGCGGTCGGACGCCCGCGAGGACGACGACGAACGTCCACTAG
ACGTTGGCGGACCCTCCAGCCCGCCGCCGCCCGCGACGCAACACACGAGCAGCTCGTGGTTCAGT
TCGAGTGGAGGCGGCGCGGACTCCGGTCCAGAGGAAGCCGGCTCGGACTCGTCGTGCTCCGGCCC
CGCGCGCGCTCCCTCGCCCCCCGCCGGGCCCTCGCGGCCTTCTCCCGCCCCTGACGTGTCTCTCGGC
CCGCCGGTGCAGCCCCCCCTCCTGCCCTACCTGTACCCGCCTTCGCTGTACCCGCCACCGTTCTTCC
CGCCACACCAAATGCCGCCAGGTCTGTTGTTCAACCTGCATCCCCTCCTGCAGCAGTACTCGTTGC
CCCCTCCCCTCGCGCCGCCGACCCCCACGTCCGCGCCTTCTCTGAGCAAGACGCACAGGTTCGCGC
CGTACGCGCTGCCCGGGTTAGGGTCTGCGTTCGAGCAGGTCGCGCCCAGAGCGAGGAGCCTCAGT
TCGTCGCCGGCGCGGCCGCGGGTGGGGTCGCCTCCCACGAGAGCGGCGTCAGCGGACCCCCCGCC
GGACGCGCCGACGTCGACCACCTCCGCCGCGACCCCTCCCGCGTCCGACCTCAAGAGCATCGAGC
GTATGGTCAACGGCTTGGACGTGGAGACGCAGGACTGA

omb1_HCR_P1B1: gAggAgggCAgCAAACggAACGAATAATCGATAGAGGCGCCACCG

omb1_HCR_P2B1: CGCGGCGCTACGCGCGACCGAGAGGTAgAAgAgTCTTCCTTTACg

omb2_HCR_P1B1: gAggAgggCAgCAAACggAACATGACGCGGCGGGCGCCACCCGCG

omb2_HCR_P2B1: GGTCGCGCCAGTCGCGCGGTGGTCGTAgAAgAgTCTTCCTTTACg

omb3_HCR_P1B1: gAggAgggCAgCAAACggAACTTTGACAACATCCGTTGACTTTGG

omb3_HCR_P2B1: ACTAGGCACTGGGTTTGGCCACAGCTAgAAgAgTCTTCCTTTACg

omb4_HCR_P1B1: gAggAgggCAgCAAACggAACAGGATCGTCAAATGATCTGAGTGG

omb4_HCR_P2B1: CCGTGGCTGGTACTTGTGCATCGAGTAgAAgAgTCTTCCTTTACg

omb5_HCR_P1B1: gAggAgggCAgCAAACggAACGTTCTGGTAGGCGGTGACGGCGAT

omb5_HCR_P2B1: TGTCGATTTTCAGCTGCGTTATCTTTAgAAgAgTCTTCCTTTACg

omb6_HCR_P1B1: gAggAgggCAgCAAACggAACCGCCCCCGTGTCTCGGAAGCCTTT

omb6_HCR_P2B1: ACACGGACAGATTCTTCTCCCGCTTTAgAAgAgTCTTCCTTTACg

omb7_HCR_P1B1: gAggAgggCAgCAAACggAAGTCGCGGGCGGCGGCGGGCTGGAGG

omb7_HCR_P2B1: CTGAACCACGAGCTGCTCGTGTGTTTAgAAgAgTCTTCCTTTACg

omb8_HCR_P1B1: gAggAgggCAgCAAACggAACTTCCTCTGGACCGGAGTCCGCGCC

omb8_HCR_P2B1: GGCCGGAGCACGACGAGTCCGAGCCTAgAAgAgTCTTCCTTTACg

omb9_HCR_P1B1: gAggAgggCAgCAAACggAAGCGGGTACAGGTAGGGCAGGAGGGG

omb9_HCR_P2B1: GGAAGAACGGTGGCGGGTACAGCGATAgAAgAgTCTTCCTTTACg

omb10_HCR_P1B1: gAggAgggCAgCAAACggAAATGCAGGTTGAACAACAGACCTGGC

omb10_HCR_P2B1: GGGCAACGAGTACTGCTGCAGGAGGTAgAAgAgTCTTCCTTTACg

knirps (kni)

>Knirps_B1_ XM_024078837.2

ATGGCTGATGGAAGTGGGAAGAGACGGGGTCATAGAAACACAGGTGCATTCAAAACACGAGACC
GGTCCAAAACCCGATACGCCTGTAAAACTACGACCGATCATTCATATATTTGGGTGGCAATAAATT
TCCCTAAGAATGGATCCATCAGTGAAGTCACACCCATTAAACTAATGGCAGATTTATTCGATCGCG
AGGAGATTTACGATGTCGGCATCTGTTTACCGACTTCGCCCAAGGTGGACAGCTTTGTGCAGATAC
AACGGCCTGCAATCGCCAGACATACCTCATTGTATGTAATAATCTGTCAGCTCGTGCTAGTGTTAA
AACACAGCTTTTATAGAAGCGGCAGCGGGCGCCGGCTCTATCGGTCATTCTTCGGGCGCTCCTACA
ACAACCTGAACTCCATCACCGAGTGCAAGAACAACGGGGAGTGCGTCATCAACAAAAAGAACCGC
ACGGCGTGCAAAGCGTGTCGACTGCGCAAATGCCTCATGGTGGGCATGTCCAAATCCGGCTCCAG
ATACGGAAGACGATCCAACTGGTTCAAGATACACTGCCTTCTGCAAGAACAGCAACAAGCCGCCC
AGGCGCACTCTCCCCCTAGAGTACCGCAGTCGCCGCACTTAGCGCCGCCTTTTCCACCTCACCTCTT
CCCTGGACTGGCGCGACCGAGAACCAAAGAGGAACTCGCTCTGTTAGGCCTCGACGATTACAAAG
CACCCTGCTCAGGATCCCCGGACTCTCAACGAAGCGGCTCATCCCCTAAACTAGACGAAAAAGCT
CGCATCACTCACCGACCGCCTGACCGCCCTCTGACGCCACCCAGAGACTCCTTCCTCCCTTTACCTT
TAGCCTTGCCGCACTTTCCACACTCACCGTTTCTCCATCCGCAGCACTTCAACCCGTTCCCGCCGAA
TCACCACCTCCTCTTCCCGCCAGGGTTCCACCCGATATACTCCAGACATCTATTAGACCACGCCGC
ACTCAGACAAGTGGCTGAAAATAACAACGACGTGAGAATCGACGACAATAACACGGAATCATCG
AAGCGCTTCTTTTTGGATGAGATTCTTAAGCAGCAACGATCTGCGCAGCCTACGCCGCAAGAAGAT
GTGATATCTGAAGCAGAATTTGTACCCACTCCGCCGGCGGAAAGGAGAACGTCGGAGTCACCTCT
GCAAGAGAATCCAATGGATCTATCCGTCAAATCTGACGGAAGATCGAGTTCGGCGCGACGGCGGT
CCGACGACAGCGAGGTGATCGCCCCGGACAACGATGACCCAGAATCCGGGAGTGACCGAGCGTCC
GCCAGTGAAGAAGAAGATTTATCGTACTCCCAAATAAAGAGGATCAAACTCCACCCTTTAGATCT
GACAACCAAAGTCTGA

Kni1_HCR_P1B1: gAggAgggCAgCAAACggAATCGTAGTTTTACAGGCGTATCGGGT

Kni1_HCR_P2B1: TTGCCACCCAAATATATGAATGATCTAgAAgAgTCTTCCTTTACg

Kni2_HCR_P1B1: gAggAgggCAgCAAACggAAGGGTGTGACTTCACTGATGGATCCA

Kni2_HCR_P2B1: ATCGAATAAATCTGCCATTAGTTTATAgAAgAgTCTTCCTTTACg

Kni3_HCR_P1B1: gAggAgggCAgCAAACggAAGAAGTCGGTAAACAGATGCCGACATC

Kni3_HCR_P2B1: TATCTGCACAAAGCTGTCCACCTTGGTAgAAgAgTCTTCCTTTACg

Kni4_HCR_P1B1: gAggAgggCAgCAAACggAATACATACAATGAGGTATGTCTGGCG

Kni4_HCR_P2B1: TAACACTAGCACGAGCTGACAGATTTAgAAgAgTCTTCCTTTACg

Kni5_HCR_P1B1: gAggAgggCAgCAAACggAATCCCCGTTGTTCTTGCACTCGGTGA

Kni5_HCR_P2B1: GTGCGGTTCTTTTTGTTGATGACGCTAgAAgAgTCTTCCTTTACg

Kni6_HCR_P1B1: gAggAgggCAgCAAACggAAACCATGAGGCATTTGCGCAGTCGAC

Kni6_HCR_P2B1: TATCTGGAGCCGGATTTGGACATGCTAgAAgAgTCTTCCTTTACg

Kni7_HCR_P1B1: gAggAgggCAgCAAACggAACGCTAAGTGCGGCGACTGCGGTACT

Kni7_HCR_P2B1: AGGGAAGAGGTGAGGTGGAAAAGGCTAgAAgAgTCTTCCTTTACg

Kni8_HCR_P1B1: gAggAgggCAgCAAACggAAAACAGAGCGAGTTCCTCTTTGGTTC

Kni8_HCR_P2B1: CAGGGTGCTTTGTAATCGTCGAGGCTAgAAgAgTCTTCCTTTACg

Kni9_HCR_P1B1: gAggAgggCAgCAAACggAAAGTCTCTGGGTGGCGTCAGAGGGCG

Kni9_HCR_P2B1: GCAAGGCTAAAGGTAAAGGGAGGAATAgAAgAgTCTTCCTTTACg

Kni10_HCR_P1B1: gAggAgggCAgCAAACggAATGAAGTGCTGCGGATGGAGAAACGG

Kni10_HCR_P2B1: GGAGGTGGTGATTCGGCGGGAACGGTAgAAgAgTCTTCCTTTACg

spalt

>spalt_B1_XM_024083373.2

ATGCCGCGCGTCAAGCCCGCCTGCGTCCGCCGCGTCTCCATCGGTGAAAGCTCGGGATCTTGTTCG
GAGGAAGATGTTGGCAATGCCATGCCGGATGAAGCGAGAGATAGGCCAGAGGCGCACATGTGTCC
ACGCTGTCAGGAACAGTTCGAAAACCTTCACGATTTCTTGTATCATAAGCGACTTTGCGATGAGAA
AGCAATGCAAATGGGTGAAGAGAGGATGCACTCCGATCCAGAGGATATGGTAGTGTCGGGGGATG
AAGAGATGGATGGTCCCAATAAACGACTAGAACAAGTCAGGAGGCATCGACAAGATGCTGAAAA
TAATAATAGTCTCGAAGACGGCGAGGCCGAAATACCTGAAGCCGACATGCCCCCCGTGGGCTGCC
GTTCCCTTTGGCAGGACACGTTACTCTTGAGGCTCTACAAAATACGAGAGTAGCGGTCGCCCAATT
CGCTGCAACAGCGATGGCAAATAATGCGAATAACGAAGCTGCTATACAAGAATTACAAGTGTTAC
ACAACACTCTATACACTTTACAGTCACAACAAGTATTTCAACTTCAGTTAATACGTCAGCTTCAGA
ATCAGTTATCTCTAACTCGACGGAAAGAAGACGATCCACACAGCCCACCGCCAAGTGAACCAGAA
CAGAATGCCCCGTCAACGCCGGCTCGATCACCGTCGCCGCCGCGTCCGCCACGGGAGCCGTCGCCT
GTTATACCCTCTCCTCCTACTAGCCAAAGTTTGCCGTCGACTCACACACATCACACACCCAAAACT
GAACAGATATCTATCCCTAAGATTCCAACTTCCTCACCATCTTTAATGACCCACCCACTTTATAGTT
CAATTTCTTCGTCATTAGCATCTTCCATCATAACAAACAATGATCCTCCACCGTCCCTAAATGAACC
AAACACACTTGAAATGCTTCAAAAACGGGCACAGGAAGTACTCGACAATGCATCACAGGGCCTTC
TAGCAAACAATCTTGCCGACGAATTAGCTTTTCGAAAATCCGGAAAAATGTCACCTTATGATGGAA
AAAGTGGTGGCCGTAACGAACCTTTCTTTAAACATCGCTGTAGATATTGTGGAAAAGTGTTCGGTA
GCGACTCTGCACTTCAAATTCACATTCGTTCTCACACAGGGGAAAGACCTTTCAAATGTAACGTCT
GTGGCTCTCGATTTACAACCAAAGGAAATCTTAAAGTTCATTTCCAAAGGCATACTTCGAAATTTC
CACATGTCAAAATGAACCCTAATCCCGTTCCAGAACATTTGGATAAATATCACCCACCGTTATTAG
CGCAATTGTCGCCGGGGCCCATTCCTGGAATGCCGCCACATCCACTTCAGTTTCCCCCAGGAGCCC
CAGCTCCCTTTCCGCCAAACTTGCCATTATACAGGCCACCGCATCACGATTTATTGCCTCCACGCCC
TCTGGGTGATAAGCCTCTCTCACATCACCCACTTTTTGCTATGCGAGAAGAACAAGACGCACCAGC
TGATCTCAGTAAACCTTCCGCACCAAGCCCTCCTCGACCCGCGTCTGATATTTTTAAGTCTGAACCT
CAAGACGAAGAGAGTCAACGAGATTCCAGTTTTGAAGAGACTGATCGTATATCACCTAAGCGAGA
AATCGAAGACAATGATATAGGACAAGATGCAGAACAAGATCGATACCCATCCACATCACCGTACG
ATGACTGCAGTATGGATTCCAAATACAGCAATGAAGATCAAATCGGCAGAGATAGTCCACACGTG
AAGCCCGATCCAGATCAACCGGAAAATCTCTCAAGTTCGGAGAGCGGGCGGAGTGCACGGGGGTC
GCCACCGTCGCCGTCGCCGTCGCCGTCGGCGCTGTCCACGCCGCCGCGTCTGCCGCACCACTCGCC
GCTGCCGTCGCCCCCGACGCCCCTGGCGGCGCTCGGCGCGCTCGGCGGATCGCCCTTCAGCCCGCT
CGGACTTGCCTTTCCTCCCGCAGTGCGCGGCAACACAACGTGTACCATCTGCTACAAGACATTCGC
CTGCAACTCGGCACTGGAGATCCACTATCGAAGCCACACCAAGGAACGGCCATTCAAGTGCACCG
TCTGCGATAGAGGCTTTTCTACCAAGAGCAGTGGCGGCGGTTGTCAGTGCGGAAGGCGTGCGCGC
GCACCCCGCCCGCCGCACGCCACTGCTTTGGACCTCTGGAACGCCTTCGTCTACCCGGGCAACATG
AAGCAGCACATGCTAACGCACAAGATCAGAGACATGCCGCCTGGTTTTGACAAGGGGCCGGGAGG
ACCTTCCGGACCCCCAAGCGAGGAAGGGCGGGACCCCAGCCCGGACAGACGGTCGTCCCCAGAAA
AGCTGGATCTGAAAAGATCACCCCCGGTGCATCCTCCACCGCCAATGTCACACCCACCTATTGACA
TGCCACCTCTACCAAAAAGACCTACAGTGCCCAGTATCCCGAGTCACCCCCCACCGTCGCGTCGTC
GAAGCACCTGTGCGGCGTGTGTCGCAAGAACTTCTCCTCATCATCAGCGCTGCAGATACACATGCG

CACGCATACCGGAGACAAACCCTTCCGATGTGCTGTCTGTCAGAAGGCGTTTACCACCAAAGGCA
ATCTTAAGGTGCACATGGGCACGCACATGTGGAGCGGCGGCGCGTCGCGGCGCGGGCGGCGCATG
TCGCTGGAGCTCCCGCCGCGGCCGCTGCACGAGCCGCACGAGCTGCTGCGGCGCCCCGACCTCTTC
TACCCCTACCTGCCGGCGCCTTTCCTCAACGGCATGCAACAGAAGCTGAACGAGATATCTGTAATA
CAGCAGAACGCCGGACAAAACGGCGTAGCTGGAAAATTCCCCGGTCTGCTCGGCTTCGGAGCGTT
CGGGGCCGGGAGACCGGGCGCCGCGTCCCCGCTCGAGAGGCCTCCCTCGCTGGAGGGGGGAGACG
AGCGACAGGCGGCGATGCGTGAGCTGGCCGAGAGGGGACGGGAGCTGGCGGAGAGGAGTCGGCA
GATGCGCGAGGAGAGCGAGCGGGAGCACTACAGGGCCGCGGGCGGACTGCCCGCGCACGCGCAC
GCGCCCAACCCCGCGCAGGCCTCGCCGCCCGCGCCGCACGCGCACCCGCACCCCCTCGCGTCGCTG
CCGCCGCCCGCGCGGACAGAAGGCCTCACCGTATAA

spalt1_HCR_P1B1: gAggAgggCAgCAAACggAAATCCGGCATGGCATTGCCAACATCT

spalt1_HCR_P2B1: GTGCGCCTCTGGCCTATCTCTCGCTTAgAAgAgTCTTCCTTTACg

spalt2_HCR_P1B1: gAggAgggCAgCAAACggAAGTGCATCCTCTCTTCACCCATTTGC

spalt2_HCR_P2B1: CGACACTACCATATCCTCTGGATCGTAgAAgAgTCTTCCTTTACg

spalt3_HCR_P1B1: gAggAgggCAgCAAACggAAATTTCGGCCTCGCCGTCTTCGAGAC

spalt3_HCR_P2B1: CCCACGGGGGGCATGTCGGCTTCAGTAgAAgAgTCTTCCTTTACg

spalt4_HCR_P1B1: gAggAgggCAgCAAACggAACGTTATTCGCATTATTTGCCATCGC

spalt4_HCR_P2B1: ACACTTGTAATTCTTGTATAGCAGCTAgAAgAgTCTTCCTTTACg

spalt5_HCR_P1B1: gAggAgggCAgCAAACggAAGTGTGGATCGTCTTCTTTCCGTCGA

spalt5_HCR_P2B1: CTGTTCTGGTTCACTTGGCGGTGGGTAgAAgAgTCTTCCTTTACg

spalt6_HCR_P1B1: gAggAgggCAgCAAACggAAGAGTCGACGGCAAACTTTGGCTAGT

spalt6_HCR_P2B1: GTTCAGTTTTGGGTGTGTGATGTGTTAgAAgAgTCTTCCTTTACg

spalt7_HCR_P1B1: gAggAgggCAgCAAACggAAAGGATCATTGTTTGTTATGATGGAA

spalt7_HCR_P2B1: TGTGTTTGGTTCATTTAGGGACGGTTAgAAgAgTCTTCCTTTACg

spalt8_HCR_P1B1: gAggAgggCAgCAAACggAAATTTTTCCGGATTTTCGAAAAGCTA

spalt8_HCR_P2B1: CCACCACTTTTTCCATCATAAGGTGTAgAAgAgTCTTCCTTTACg

Optix

>Optix_B2_XM_024080404.2

ATGCGCGGCTCCTGGGACGAGTCCACGACGGCGGCGCTGCACGCGCGCATCCTGGAGGCGCACCG
CGGGTCCGCCGCGCCCGACCGCGCCGAGCCCGCGTGCGAGCCTCCGCCGCTGACGCTGGGCGCGC
TGGAGCTGGCGGCGCCCACGCCGCTGCTGCCGCTGCCCACGCTGAGCTTCAGCGCCGCGCAGGTG
GCCACCGTGTGCGAGACGCTGGAGGAGAGCGGCGACGTGGAGCGCCTGGCGCGCTTCTTGTGGTC
GCTGCCCGTGGCGCACCCCAACGTGGCCGAGCTGGAGCGCTGCGAAGCCGTGCTGCGCGCGCGCG
CCGTCGTCGCCTTCCACGCCGGCCGCCACCGCGAGCTGTACGCCATCCTCGAGCGCCACCGCTTCC
AGCGCTCCAGCCACGCCAAGCTGCAAGCGCTGTGGCTGGAGGCGCACTACCAGGAGGCTGAGCGC
CTGCGCGGCCGTCCGCTGGGCCCCGTCGACAAGTACCGCGTGCGGAAGAAGTTCCCGCTCCCGAG
GACGATCTGGGACGGCGAGCAGAAGACGCACTGTTTCAAGGAGCGGACGCGATCTCTACTCCGAG
AATGGTACCTCCAAGATCCCTACCCGAACCCGACGAAGAAGAGGGAATTGGCGGCGGCGACGGGT
CTGACGCCGACGCAAGTCGGCAACTGGTTCAAAAACCGACGGCAAAGAGACCGAGCGGCCGCCG
CCAAGAACCGCTCCGCCGTGCTGGGCAGAGGATAA

Optix1_HCR_P1B2: CCTCgTAAATCCTCATCAAACGCCGTCGTGGACTCGTCCCAG

Optix1_HCR_P2B2: CGCCTCCAGGATGCGCGCGTGCAGCAAATCATCCAgTAAACCgCC

Optix2_HCR_P1B2: CCTCgTAAATCCTCATCAAAGGCTCGGCGCGGTCGGGCGCGGCGG

Optix2_HCR_P2B2: AGCGTCAGCGGCGGAGGCTCGCACGAAATCATCCAgTAAACCgCC

Optix3_HCR_P1B2: CCTCgTAAATCCTCATCAAAGGCAGCAGCGGCGTGGGCGCCGCCA

Optix3_HCR_P2B2: GCGGCGCTGAAGCTCAGCGTGGGCAAAATCATCCAgTAAACCgCC

Optix4_HCR_P1B2: CCTCgTAAATCCTCATCAAACCGCTCTCCTCCAGCGTCTCGCACA

Optix4_HCR_P2B2: AAGAAGCGCGCCAGGCGCTCCACGTAAATCATCCAgTAAACCgCC

Optix5_HCR_P1B2: CCTCgTAAATCCTCATCAAAGCGGTGGCGGCCGGCGTGGAAGGCG

Optix5_HCR_P2B2: GTGGCGCTCGAGGATGGCGTACAGCAAATCATCCAgTAAACCgCC

Optix6_HCR_P1B2: CCTCgTAAATCCTCATCAAAACAGCGCTTGCAGCTTGGCGTGGCT

Optix6_HCR_P2B2: CAGCCTCCTGGTAGTGCGCCTCCAGAAATCATCCAgTAAACCgCC

Optix7_HCR_P1B2: CCTCgTAAATCCTCATCAAAACAGTGCGTCTTCTGCTCGCCGTCC

Optix7_HCR_P2B2: GAGTAGAGATCGCGTCCGCTCCTTGAAATCATCCAgTAAACCgCC

Optix8_HCR_P1B2: CCTCgTAAATCCTCATCAAACGTCGGGTTCGGGTAGGGATCTTGG

Optix8_HCR_P2B2: CGTCGCCGCCGCCAATTCCCTCTTCAAATCATCCAgTAAACCgCC

Optix9_HCR_P1B2: CCTCgTAAATCCTCATCAAATTTGAACCAGTTGCCGACTTGCGTC

Optix9_HCR_P2B2: GGCCGCTCGGTCTCTTTGCCGTCGGAAATCATCCAgTAAACCgCC

thickvein (tkv)

>thickvein_B2_XM_024079796.2

ATGATGTACTGTACCAGCCCTGCGGCTACATCTGGCTGCGTGTCCGTCATCATGGCCGGTGGCCAA
GTTTGCCTAGGTCGGGGCATCGTGTGCGAGTGCACGGGCGCGGGCATGTGCCCCGGCGGCGCGCC
CAACGGCACGTGCGGCACGCAGCCCGGCGGGTACTGCTTCGTGGCCGTGGAGGAGCTCTACGACG
ACAGCGGGCTCGTGGTGCTGGAGCGCACCGCCGGCTGCCTGCCGCCCGACGAGTCGGGCCTCATG
CAGTGCAAGAAAGTGCCCCACCAGAACCCGAAGGCGATAGAGTGCTGCGAGAAAGACTACTGCA
ACCGCCGCCTGCGGCCGCAGCTGCCGGAGCCCCCGCCCGACGTCACCGAGACCCCCGGCCTGCGC
CCCGCGGGCTCCGTGCCGCACACGGCGCTGGTCGCCGCGGCGCTGTGCGCGGCCCTGCTCGCCTTC
CTCGCGGCCTTCTGGCTGCTCTTCAGGATGCGCAGAAGAGGATGCAAGCGACCGCCTTCCCCGCCC
GCCCCCGCGCACAGCTCGGAGATCTCCTCGGGCTCCGGGTCCGGCCTCCCGCTCCTAGTCCAAAGA
ACCGTCGCCAAACAGATACAAATGGTCGAGTCGATCGGCAAAGGTCGCTACGGCGAAGTCTGGTT
GGCGAGATGGCGCGGCGAAAAGGTGGCCGTCAAAGTTTTCTTCACCACGGAGGAGGCTTCCTGGT
TCCGCGAGACGGAGATATACCAGACGGTTCTCATGCGACACGAAAACATCCTCGGCTTCATCGCG
GCGGACATCAAAGGAACGGGATCCTGGACTCAGATGCTTCTCATCACGGACTACCACGAGAACGG
CTCCCTGCACGATTATTTGCAGACCGTCGTTCTGGACACGCAGGGTTTGATGACGATGGCGTACTC
CATAGTGAGCGGGCTGGCCCACCTGCACATGGACATATTCGGCACCAAAGGCAAGCCCGCCATCG
CTCACAGAGACATAAAGAGCAAAAACATCCTCGTCAAAAGGAACGGCCAGTGCGCGATCGCCGAC
TTCGGCCTCGCGGTCAGATACGTGGCGGAGAGGAACGAGGTGGACATCGCACCGAACACGCGCGT
CGGCACGAGGCGGTACATGGCGCCCGAGGTGTTGGACGAGAAGTTGGACGTCACCAACTTCGAGG
CGTTCAAAATGGCCGACATGTATTCTTTGGGACTGGTGATGTGGGAGATGTGCAGGCGGTGTACGA
CCGGGGACAAGGCGCAGTACGTGGAGGCGTACGCGCTACCGTACCACGAGCACGTGCCGTCGGAC

CCGTCGTTCGACGACATGCACGCGGTGGTGGTGGGCCAGCGCGCACGGCCGCCGCTGCCGGCGCG
CTGGCGGGCGTCGCCCACGCTGCTGGCGCTGGCGGCGCTCATGGCGGAGTGCTGGCACCACAACC
CGCCCGTGCGGCTCACGGCGCTGCGCGTCAAGAAGACGCTGGCCAAGTTCCGCGCCGAGAGCGCC
GTGAAGCTCGTCTGA

tkv1_HCR_P1B2: CCTCgTAAATCCTCATCAAACGTGCACTCGCACACGATGCCCCGA

tkv1_HCR_P2B2: CGCGCCGCCGGGGCACATGCCCGCGAAATCATCCAgTAAACCgCC

tkv2_HCR_P1B2: CCTCgTAAATCCTCATCAAAGCGGTGCGCTCCAGCACCACGAGCC

tkv2_HCR_P2B2: CCCGACTCGTCGGGCGGCAGGCAGCAAATCATCCAgTAAACCgCC

tkv3_HCR_P1B2: CCTCgTAAATCCTCATCAAAGGGGCTCCGGCAGCTGCGGCCGCAG

tkv3_HCR_P2B2: GGCCGGGGGTCTCGGTGACGTCGGGAAATCATCCAgTAAACCgCC

tkv4_HCR_P1B2: CCTCgTAAATCCTCATCAAACTGCGCATCCTGAAGAGCAGCCAGA

tkv4_HCR_P2B2: GGGGAAGGCGGTCGCTTGCATCCTCAAATCATCCAgTAAACCgCC

tkv5_HCR_P1B2: CCTCgTAAATCCTCATCAAACGATCGACTCGACCATTTGTATCTG

tkv5_HCR_P2B2: ACCAGACTTCGCCGTAGCGACCTTTAAATCATCCAgTAAACCgCC

tkv6_HCR_P1B2: CCTCgTAAATCCTCATCAAATCGCATGAGAACCGTCTGGTATATC

tkv6_HCR_P2B2: CGCGATGAAGCCGAGGATGTTTTCGAAATCATCCAgTAAACCgCC

Mother against decapentaplegic 6 (Mad6)

>Mother_against_decapentaplegic_homolog_6 (Mad6)_Bany_10682-RB_B1

ATTTCAGAACACGGCGAAATTACACAAGACGCGAGGAGGACGAATCATGAGCGGCTTGCTACAGG
CTCCCTCGCTACTGATGGCGAAGAGCGGCAGAGCTGGGAGACCGAGTGGTGCAGGCTGGCGTACT
GGGAGCTGACGCAGCGTGTGCGAAAAACTCGAGCGAAGATTGGCCTAGGTGTCACACTGTCCTTA
GAATCTGATGGCGTCTGGCTCTACAATAGAAGCCAAGAACCCGTGTTCGTCAGCTCCCCCGCGTTA
GACGCTGCTGCTGCGAAAGCTCTTCTTGTATGGAGGGTTGCACCAGGACACTGTCTCTGCATCTTC
GACCCCTCGTCGCCCCCGCCCGCTGTGTCGCTACCCCACGTGGGGCCAGTTGACCCCAGATCTGTG
AGGATATCGTTCGCGAAAGGCTGGGGCCCCAAATACTCGAGGCGTGACGTCACCGCCTGCCCCTG
TTGGCTCGAAGTCCTGCTGGCGCCTCCGAGCTGA

Mad61_P1_B1	gAggAgggCAgCAAACggAACGTCTTGTGTAATTTCGCCGTGTTC
Mad61_P2_B1	CAAGCCGCTCATGATTCGTCCTCCTTAgAAgAgTCTTCCTTTACg
Mad62_P1_B1	gAggAgggCAgCAAACggAACCCAGCTCTGCCGCTCTTCGCCATC
Mad62_P2_B1	AGTACGCCAGCCTGCACCACTCGGTTAgAAgAgTCTTCCTTTACg
Mad63_P1_B1	gAggAgggCAgCAAACggAAGCCAATCTTCGCTCGAGTTTTTCGC
Mad63_P2_B1	AGATTCTAAGGACAGTGTGACACCTTAgAAgAgTCTTCCTTTACg
Mad64_P1_B1	gAggAgggCAgCAAACggAACAGAGACAGTGTCCTGGTGCAACCC
Mad64_P2_B1	GGCGGGGGCGACGAGGGGTCGAAGATAgAAgAgTCTTCCTTTACg
Mad65_P1_B1	gAggAgggCAgCAAACggAATCACAGATCTGGGGTCAACTGGCCC
Mad65_P2_B1	GGCCCCAGCCTTTCGCGAACGATATTAgAAgAgTCTTCCTTTACg

Mother against decapentaplegic 3 (Mad3)

>XM_024082355.2 PREDICTED: *Bicyclus anynana* mothers against decapentaplegic homolog 3 (LOC112045951), transcript variant X2, mRNA

ATGCAGAGCGTGGTGGGCGTCGTGGGCGCGGTGGGCGGCCACAGCGCGCTGTATCTGGAGGCCAC
GCTGGCGCAGCAGGTGCCCGGCAACACCACCACGCCGCCGCCGGGCTACATGAGCGAGGACGGCG
ACCCCATGGACCACAACGACAACATGAACCTGACCCGCCTGACCCCGTCCCCCGGGAACATAGCG
ACGGAGGCGGCGCCCGTGCTGTACCACGAGCCCGCCTTCTGGTGCAGCATCAGCTACTACGAGCT
GAACACGCGCGTGGGAGAGACCTTCCATGCTTCACAACCCTCGATCACCGTGGACGGCTTCACGG
ATCCCAGCAATAGTGAAAGGTTTTGCCTGGGCCTGCTGTCGAACGTGAACAGAAACGAGGTGGTG
GAGCAGACGCGCCGACACATCGGCAAGGGCGTGCGGCTCTACTATATCGGCGGCGAGGTGTTCGC
GGAGTGCCTCAGCGACTCGGCGATATTCGTGCAGAGCCCCAACTGCAACCAGCGGTACGGCTGGC
ACCCCGCCACCGAGTTCGCGGCGCTGCTGTCGCAGTCGGTGTCGCAGGGCTTCGAGGCGGTGTTCC
AGCTCACGCGCATGTGCACCATCCGGATGAGCTTCGTCAAGGGCTGGGGCGCCGAGTACAGGCGG
CAGACGGTGACGTCGACGCCATGCTGGATCGAGCTGCACCTGAACGGGCCGCTGCAGTGGCTGGA
CCGCGTGCTCACGCAGATGGGCTCGCCGCCGCTGCCGTGCTCCTCCATGTTAAACCACAGAGTCTA
TCGGTCAAAGAAAATGAAAAAAAAAAATGAGCACAGAACTTATAAATAG

Mad31_P1_B2	CCTCgTAAATCCTCATCAAAGCGGCGTGGTGGTGTTGCCGGGCAC
Mad31_P2_B2	CGCCGTCCTCGCTCATGTAGCCCGGAAATCATCCAgTAAACCgCC
Mad32_P1_B2	CCTCgTAAATCCTCATCAAATGATGCTGCACCAGAAGGCGGGCTC
Mad32_P2_B2	CCACGCGCGTGTTCAGCTCGTAGTAAAATCATCCAgTAAACCgCC
Mad33_P1_B2	CCTCgTAAATCCTCATCAAACTCCACCACCTCGTTTCTGTTCACG
Mad33_P2_B2	CGCCCTTGCCGATGTGTCGGCGCGTCAAATCATCCAgTAAACCgCC
Mad34_P1_B2	CCTCgTAAATCCTCATCAAAAACTCGGTGGCGGGGTGCCAGCCGT
Mad34_P2_B2	GACACCGACTGCGACAGCAGCGCCGAAATCATCCAgTAAACCgCC
Mad35_P1_B2	CCTCgTAAATCCTCATCAAAATCCAGCATGGCGTCGACGTCACCG
Mad35_P2_B2	TGCAGCGGCCCGTTCAGGTGCAGCTAAATCATCCAgTAAACCgCC

Mother against decapentaplegic 4 (Mad4)

>Motherss_against_decapentaplegic_homolog_4 (Mad4) _Bany_17230-RA_B3
ATGAACACTACAGCACCGACATCGGCAGATGCCTGCCTCAGTATAGTCCACTCCCTGATGTGCCAC
AGGCAAGGGGGTGAGAGCGAGGGTTTCTCCAAGAGAGCTATTGAGTCACTAGTCAAGAAGTTGAA
GGAAAAGAGAGATGAATTGGACTCCTTGATCACAGCCATCACCACTAACGGTGCTCACCCTAGCA
AATGTGTCACTATACAACGCACATTAGATGGTCGGTTACAGGTAGCAGGAAGAAAGGGTTTTCCTC
ACGTCATCTACGCCCGTATATGGCGCTGGCCGGATCTCCACAAAAATGAACTGAAACATGTGAAG
TTTTGTCAGTTTGCATTTGATCTCAAGTGTGACTCAGTGTGCGTTAACCCTTACCACTATGAGAGAG
TTGTGTCTCCAGCGCCAGATGGCGTTGCGAATATGTTTTCCCCACACGGCCCGCGGCCCTCGATGC
GACCGGGAGCGCCATTGGTGCCGCCACAAATGGTGCACTCGCCTGGCGCGCAAATGCTAAACCAC
CAGTCGAATCAGATGCCAGGCACACCGCAAATGGGGCCGGGGACACCGCAAATGGGTCCGGGGA
CACCGCAAATGGGACCGGGTACACCACAAATGGGGCCGGGCACACCGCAGATGGGGCCGGGCAC
ACCGCAAATGGGACCAGGGCCGCCTCAGATGGGGCCGGGGACACCGCAGATGGGGACAAACGTG
CCGCAGATGGCGTCCCCGAGAATGGCGTCGGCTCCCACACAGATGTCCCCGGGGACGCCCCAGAT
GCCGAGTATAAGCCAGGGCATGTCTATATCGAGCCCCCAACAAATGGCAATGGCGCAACAGAGAG
CTATACTCGCCCCAAAACTGGAACCGCCCGATACAATGGACGCGCGCGCGATGTACTCCCCGGGG
TGCCGAGATGTGACGGGCGAGGCGGGGGAGGCGGTACATGGAGCCGGCTGGCGGATTGACGAAG
ATGGTGACGCGCAGATGCGTACATGGACAGGCAACAACACACTAACATACACGCAGAGCTTGGCG
CCGCCGCCGGCCGCCCCGCCCGTGCCGGTTGATGCGCCTGCGCATCATCATCATTATTATAATGGT
AATCCTGGTGGTTTGCTGTCGAGTCAACCAGCCCCAGAGTATTGGTGCTCAGTGGCATACTTTGAA
CTGGACACACAAGTGGGAGAGACATTCAAAGTGCCTTCTAGTAGGCCCAATGTTACTGTTGACGGT
TACGTTGATCCTTCTGGCGGCAACAGATTTTGTTTAGGAGCCCTAAGTAATGTACACAGGACAGAG
CAGAGTGAACGAGCTAGGCTCCATATCGGTAAAGGCGTTCAGCTGGACCTGCGCGGCGAGGGCGA
CGTGTGGCTACGGTGTCTCTCAGACCACTCGGTGTTCGTGCAGTCGTACTACCTCGACCGGGAGGC
GGGCAGGGCGCCGGGCGACGCTGTGCACAAGATATACCCGTCGGCTTGTATTAAGGTATTCGACC
TCCGGCAGTGCCACCGTCAGATGCAGACGCAGGCGCGCACGGCGCAGGCCGCCGCCGCCGCGCAG
GCCGCCGCCGTCGCAGGACACATACAGCCTGCGCATCCTGGGATGAACAAATGTCTATCAGCTGC

CGCCGGCATCGGTGTAGACGACTTGCGGCGGCTCTGCATCGTGCGACTGTCGTTTGTGAAGGGCTG
GGGACCCGACTACCCTCGGACCTCCATCAAAGAGACCCCCTGCTGGGTTGAAGTACATTTACACAG
AGCATTGCAACTTCTGGACGAGGTGTTGCATACGATGCCCATCGACGGTCCTCGGACGAGCATCGA
GTAG

Mad41_P1_B3	gTCCCTgCCTCTATATCTTTGCTCTCTTGGAGAAACCCTCGCTC
Mad41_P2_B3	TTCAACTTCTTGACTAGTGACTCAATTCCACTCAACTTTAACCCg
Mad42_P1_B3	gTCCCTgCCTCTATATCTTTTTCTTCCTGCTACCTGTAACCGACC
Mad42_P2_B3	GGGCGTAGATGACGTGAGGAAAACCTTCCACTCAACTTTAACCCg
Mad43_P1_B3	gTCCCTgCCTCTATATCTTTACAACTCTCTCATAGTGGTAAGGGT
Mad43_P2_B3	TTCGCAACGCCATCTGGCGCTGGAGTTCCACTCAACTTTAACCCg
Mad44_P1_B3	gTCCCTgCCTCTATATCTTTCTGGCATCTGATTCGACTGGTGGTT
Mad44_P2_B3	GTGTCCCCGGCCCCATTTGCGGTGTTTCCACTCAACTTTAACCCg
Mad45_P1_B3	gTCCCTgCCTCTATATCTTTCCCATCTGAGGCGGCCCTGGTCCCA
Mad45_P2_B3	TTTGTCCCCATCTGCGGTGTCCCCGTTCCACTCAACTTTAACCCg

abrupt

>lcl|XM_024085182.2_cds_XP_023940950.1_1 [gene=LOC112047886] [db_xref=GeneID:112047886] [protein=protein abrupt isoform X2] [protein_id=XP_023940950.1] [location=231..1376] [gbkey=CDS]

ATGGCAATGCCTGAGCAGTTTTCATTGCGTTGGAATGATTTCCACGCAAACTTGTCCCAGTCTTTCC
AAGCTTTGTTGGAGGGCGAAGACCTAGTAGACGTGACGCTGGCGGCTGGTGGTCAGTATGTCCAC
GCTCACAAGCTCATCCTCTCAGTATGCAGTCCCTATTTCAAGGAGCTATTTAAGATGAATCCCTGC
GAGCACCCAATAGTAATACTAAAGGATGTAGCTCACCAGGAATTGCGACAGCTTTTGCAATTTATG
TACCGTGGAGAAGTCCACGTCAGACAGCAAGAGTTGTCTGCTTTTCTTCACACAGCAGAGCTGCTG
CAAGTCAAAGGGCTTACGGGCGGTAGAGAGAGAAGTGAATCACCTCCTGCACCAGTGGAAGAAG
ATATACTAAATGCTCCTATGCCACCTGAACCTATTGGTGATAGTTTGCCTGAATGGGTACCATCAA
ATGATGAAGCGATCCCTCCTGAAGTCTCGCAAGAATCTGCTAGCTCTCTAGTCCCTAAAGACGAAG
CTGCTCGGAGCCCTTTGAAAAGGTTGTTGAAGAACACTACAAACAAAAACAGTTACAACATCAAG
AAGAAACCACGGCCCGTGAGCGATAATAGCCCTTCGCTTGCTGAAAACACGGAATACTCCTCTGA
CGGTGAATTAATGATCGACTTTGACAACGATATGCTTCACAATCTGGTTTCGGCAGATTCAGCCAA
AGAGTCCGGATGGAACTGCAAAACGGGCGGGGTTAAATGTCCGTCTTGTCACCGATTTTTCGCGAA
TCGATACAATCTGAAAGTACATATTCGGGACAAGCACGACACAAGAGAAGGAACTCTGCAGTGTG
ACATTTGTCAGAAACGTATGCGCAACCCATCGTGTCTCCGTGTCCACATGTACCATCATCGCAAGC
AGGCCGCCTACTTGGCACAGCTCAGTGCCCAAGGAGACCAAATGAGTGTACAAAACATGGTTGGA
AATAAATGGCGCCCGGATCCCAACACTGAACTGCGAGATGTCGATAATTACCCAGCGTCAGGAGC
CATGGAACTGAAGTACCAGCCGGCTGGCGACGCCCCGTTGCCGGCAGTGGCCGAGGCAACGCTTC
CGAAAGTGGGAGCAAATGTGGAGACGGCATGA

B2_P1 (abrupt)_1 CCTCGTAAATCCTCATCAAAAAATAGGGACTGCATACTGAGAGGA

B2_P1 (abrupt)_2 CCTCGTAAATCCTCATCAAATTCCTGGTGAGCTACATCCTTTAGT

B2_P1 (abrupt)_3 CCTCGTAAATCCTCATCAAAGAAAAGCAGACAACTCTTGCTGTCT

B2_P1 (abrupt)_4 CCTCGTAAATCCTCATCAAAGCAGGAGGTGATTCACTTCTCTCTC

B2_P1 (abrupt)_5 CCTCGTAAATCCTCATCAAACCATTCAGGCAAACTATCACCAATA

B2_P1 (abrupt)_6 CCTCGTAAATCCTCATCAAACTTTAGGGACTAGAGAGCTAGCAGA

B2_P2 (abrupt)_1 CAGGGATTCATCTTAAATAGCTCCTAAATCATCCAGTAAACCGCC

B2_P2 (abrupt)_2 GTACATAAATTGCAAAAGCTGTCGCAAATCATCCAGTAAACCGCC

B2_P2 (abrupt)_3 TGACTTGCAGCAGCTCTGCTGTGTGAAATCATCCAGTAAACCGCC

B2_P2 (abrupt)_4 GCATTTAGTATATCTTCTTCCACTGAAATCATCCAGTAAACCGCC

B2_P2 (abrupt)_5 AGGGATCGCTTCATCATTTGATGGTAAATCATCCAGTAAACCGCC

B2_P2 (abrupt)_6 TTTTCAAAGGGCTCCGAGCAGCTTCAAATCATCCAGTAAACCGCC

Bicyclus anynana testis-specific gene A8 protein-like (A8)

>lcl|XM_052885974.1_cds_XP_052741934.1_1 [gene=LOC128198825] [db_xref=GeneID:128198825] [protein=testis-specific gene A8 protein-like] [protein_id=XP_052741934.1] [location=46..786] [gbkey=CDS]

ATGAACTCGCTGGTGGTGTTGTTATCCGTGATGGCGCTGGCCTCTGCCAAGCCCGGCTTCCCATTTA
TCATCGACTACGCTGCGCCGGCTTTGGCGCTGGCTCCGGCAGCGGTTTCCCACCAGTCGAGGATAG
ACGTCAAATCTACGCCAGCTATTGTCAAGACTGACATTGTAGCTCCAGCAATCGCACCTGTAGTGA
CAGCGCCTATCGCTTACTCAGCGCCATTAGCTGTCGCCCCCGCCGCAGTGTCGACTCAATCACGCA
TCGATATCAAGTCATCTCCGGGTGTAATCAGCACATACGCCGCTGGACCCCTGGAGTACACTGCGC
CCCTGGCATACAGCCACGGTATCGGTTATGGTGCTCCCATATTAGCGTACAGTCCATCCATCTACG
CTGCCGCCATCCCACCGATCGGCCTGAAGTCCATCGCTATCCCATCTCAAGCGGAGCCTGCACCTG
AAAGCCCTGCGGCCCCTGAAGCTGGTTTACCGGTTGCGCCTACTGAAACTCCTGAGGTAGCCGCCG
CGCGCGCCGCTCATCTAGAAGCTAAAGCCCTGGAGGAATCCCATCAGATCCAGAAACGCTCCGTT
GGCATCCTCACTTCGCCTGTGATTTCTTCCCCGATCATAACCCGCTACGCATCTCCCGTCATCTACT
CATCCCCTATTGCGAGAATCTCCCATGTGGGTGTACCGACTCCTATCCTGACGGGTGCCTATGGAC
TCCACCCCTACTAA

B2_P1 (A8)_1 CCTCGTAAATCCTCATCAAAATGTCAGTCTTGACAATAGCTGGCG

B2_P1 (A8)_2 CCTCGTAAATCCTCATCAAAGGGGGCGACAGCTAATGGCGCTGAG

B2_P1 (A8)_3 CCTCGTAAATCCTCATCAAACAGCGGCGTATGTGCTGATTACACC

B2_P1 (A8)_4 CCTCGTAAATCCTCATCAAATACGCTAATATGGGAGCACCATAAC

B2_P1 (A8)_5 CCTCGTAAATCCTCATCAAACGCTTGAGATGGGATAGCGATGGAC

B2_P1 (A8)_6 CCTCGTAAATCCTCATCAAACCTCAGGAGTTTCAGTAGGCGCAAC

B2_P2 (A8)_1 ACTACAGGTGCGATTGCTGGAGCTAAAATCATCCAGTAAACCGCC

B2_P2 (A8)_2 GATGCGTGATTGAGTCGACACTGCGAAATCATCCAGTAAACCGCC

B2_P2 (A8)_3 CCAGGGGCGCAGTGTACTCCAGGGGAAATCATCCAGTAAACCGCC

B2_P2 (A8)_4 ATGGCGGCAGCGTAGATGGATGGACAAATCATCCAGTAAACCGCC

B2_P2 (A8)_5 GGCCGCAGGGCTTTCAGGTGCAGGCAAATCATCCAGTAAACCGCC

B2_P2 (A8)_6 CTAGATGAGCGGCGCGCGCGGCGGCAAATCATCCAGTAAACCGCC

heat shock protein 67B1-like (hsp67B1)

>lcl|XM_024094531.2_cds_XP_023950299.2_1 [gene=LOC112054669] [db_xref=GeneID:112054669] [protein=heat shock protein 67B1-like] [protein_id=XP_023950299.2] [location=57..1211] [gbkey=CDS]

ATGTCGCGGTATTTTGCGTTCTTCGCGCTCGTCGCGCTCGCGGCGGCCTACCCGGCCAGCGACGACTTCC
CTCGCCCCATCACAACAATAACTGAATCGGACTTCGAAGACCAATCGCCTTGGTTCCGATTCCCACCTTT
CGGAAACATCTTCGCGCCGCTAACGAAACTGTTCTCAAGTTTTGCGGAGATCGGACCGAAGATCGAAATT
GACGAAGACAAATTCCGTGTCATCGTAAACGTTAAGGATTACAAGAAGAAAGATCTGAAAGTTAAAGTGA
AAGGTGATTACATCCTCGTCCAAGGAGCGCACGAGGCTAAGCATGACGACCACGACTTGTTCGCGAGCCA
ATTCTTCCATACGTACAGCCTTCCGTTGAATGCTAGTGCATCAGATGTCACTGCTACTTTGTCTAGCGAT

GGATATTTGGATGTAACCGCTCCTGTGAATGGTGTCGATGACAAGAACAAGGTTGTTGATAGAGAAGTGC
CAATAGTTGAAAGTGGCAAGCCGTTGAAAGAGGATAAAGAAGATAGAGAACCTGTTGTACCCGTCGCTAG
TGCAGACCCAGTTGAAACTGTTGATAAGACAGAAAATGTTGATAAAATCGAAACTTTTGACACGCCAGTA
GAACCTTTAGCTAAGGTCGAAAATGCTGATAAAGTTGAAAATCTTGAACCACCCGTGGAACCGAGTGCCA
AAGCTGATAATGTTGATACGATTGAAGTTCCAAATGCACCAGTAGAACCTCTAGCTAAGGTCGAAAATAT
TAATAAAGTTGAAAATCGTATCGCGCCAGAGGAACCTATTACCAATGTTGAAAATGTTGATAAAGTTGAA
TCTCCCGCTGTACCAGAGGAGCCAGTGGCAAAGGTTGAAAATGTTGACAAAGTTGAAACTCCCGATGCAC
CAGTGGTGCCTGTTGCCAAGGATGAAAATGTCGACAATGTTGAAACTCCCGACGTATCAGTAGAACCTCT
ACCTAAGGTCGATAATGTCGATAAAGTCGAACAACTCCCCGAAGTGACAACTGCAACAGCTTCTGAAGGT
GAAGAAAAGACAGAGGCCCCGACGACCCAAGCGCCAGCACGTGAGGAGGTAAAAGAAGACATCAAAGTCC
CCCAGGACAACGAAGTAAACGAAATCCAGCCCTAA

B1_P1 (hsp67B1)_1 GAGGAGGGCAGCAAACGGAAGTTTACGATGACACGGAATTTGTCT

B1_P1 (hsp67B1)_2 GAGGAGGGCAGCAAACGGAAGCGCTCCTTGGACGAGGATGTAATC

B1_P1 (hsp67B1)_3 GAGGAGGGCAGCAAACGGAATTCAACGGAAGGCTGTACGTATGGA

B1_P1 (hsp67B1)_4 GAGGAGGGCAGCAAACGGAAATTCACAGGAGCGGTTACATCCAAA

B1_P1 (hsp67B1)_5 GAGGAGGGCAGCAAACGGAACTTTCAACGGCTTGCCACTTTCAAC

B1_P1 (hsp67B1)_6 GAGGAGGGCAGCAAACGGAAGTCTTATCAACAGTTTCAACTGGGT

B1_P2 (hsp67B1)_1 CAGATCTTTCTTCTTGTAATCCTTATAGAAGAGTCTTCCTTTACG

B1_P2 (hsp67B1)_2 AGTCGTGGTCGTCATGCTTAGCCTCTAGAAGAGTCTTCCTTTACG

B1_P2 (hsp67B1)_3 GTAGCAGTGACATCTGATGCACTAGTAGAAGAGTCTTCCTTTACG

B1_P2 (hsp67B1)_4 AACAACCTTGTTCTTGTCATCGACATAGAAGAGTCTTCCTTTACG

B1_P2 (hsp67B1)_5 CAACAGGTTCTCTATCTTCTTTATCTAGAAGAGTCTTCCTTTACG

B1_P2 (hsp67B1)_6 AAAGTTTCGATTTTATCAACATTTTTAGAAGAGTCTTCCTTTACG

muscle-specific protein 20-like (msp20)

>lcl|XM_024099043.2_cds_XP_023954811.1_1 [gene=LOC112058319] [db_xref=GeneID:112058319] [protein=muscle-specific protein 20] [protein_id=XP_023954811.1] [location=134..682] [gbkey=CDS]

ATGCCTGGACGTCCTATCTGGCAGTGTGCCAACAAACGTGAGCCTGAAAAGGAGCAGGAGGCCCAGAAGT
GGATCGAAGCGGTCATCGGAGAGAAGTTCCCCGCAGGCGTACCTTACGAGCATGCCCTTCGCGACGGCAT
CATCCTCTGCAAGCTGATGAACAGGCTCCAGCCCGGCATCATCTCTAAAGTGAACATCTCCGGCGGCGAC
TACAAGTTCATGGACAATATTAGCCAATTCCAAAAAGCGTGTATCAAATACGGAGTTCCAGACACAGATC
TCTTCCAGTCAACTGACCTCTGGGACCAAAAGAACATCGCACTGGTGACACAAACGATCTTTGCTCTTGG
AAGAACGTCATACAAGCACCAAGAATGGCGTGGTCCTTTCCTTGGCCCCAGGCCTGCTGAAGAAAATCGC
CGCGAATTCAGCGAGGACGTCCTCAGGGCAGGAGAGGCAGTTATCGGCTTACAAGCCGGTACAAACAAAA
TGGCGTCCCAGTCTGGACAAAACTTTGGTGCTTCGCGCAAGATTATTCTCGGCAAGTGA

B2_P1 (msp20)_1 CCTCGTAAATCCTCATCAAAGGAACTTCTCTCCGATGACCGCTTC

B2_P1 (msp20)_2 CCTCGTAAATCCTCATCAAATGGAGCCTGTTCATCAGCTTGCAGA

B2_P1 (msp20)_3 CCTCGTAAATCCTCATCAAAGAATTGGCTAATATTGTCCATGAAC

B2_P1 (msp20)_4 CCTCGTAAATCCTCATCAAATTTGGTCCCAGAGGTCAGTTGACTG

B2_P1 (msp20)_5 CCTCGTAAATCCTCATCAAACGCCATTCTTGGTGCTTGTATGACG

B2_P1 (msp20)_6 CCTCGTAAATCCTCATCAAATGCCCTGAGGACGTCCTCGCTGAAT

B2_P2 (msp20)_1 GGGCATGCTCGTAAGGTACGCCTGCAAATCATCCAGTAAACCGCC

B2_P2 (msp20)_2	ATGTTCACTTTAGAGATGATGCCGGAAATCATCCAGTAAACCGCC
B2_P2 (msp20)_3	AACTCCGTATTTGATACACGCTTTTAAATCATCCAGTAAACCGCC
B2_P2 (msp20)_4	TCGTTTGTGTCACCAGTGCGATGTTAAATCATCCAGTAAACCGCC
B2_P2 (msp20)_5	GCAGGCCTGGGGCCAAGGAAAGGACAAATCATCCAGTAAACCGCC
B2_P2 (msp20)_6	GGCTTGTAAGCCGATAACTGCCTCTAAATCATCCAGTAAACCGCC

lebercilin-like protein (lebercilin)

>lcl|XM_052882629.1_cds_XP_052738589.1_1 [gene=LOC112058222] [db_xref=GeneID:112058222] [protein=lebercilin-like protein] [protein_id=XP_052738589.1] [location=1..1833] [gbkey=CDS]

ATGTCAGAGCTTAGCCTCGTCCCAGAAGAGAAACGACGAGATCAAAGTTTGTATTGCAGGGACTCACTGG
AGAGCGTGTACAGTAGCAACTCCCGCCTCAACCTCCTGCACAAGCGCAAGCGACTCAACCTGTGCGTGGG
CATGTCCAACCAGTCGCTCAACCACAAGGGTGACAGATACGTCACCCAGAGGGTGCTCTCCGCCAAGACC
CATAGGGTGAAGCAGCTGCAGAATCAGTTGGCTGATGCGCACTATCATTTACAAGAACTTAGCAACGAAA
ATCGAGTGTTACGTGCTCTCCAAAAGAAACAAGAAATCGCGTTACAGAGATACGAGAACTCGAACGCGGA
GCTGCCCCAAGTGCTGAACTCCCACAGCGAGGAGATGCGCGTGCAGCAGAGCCGGTACAAGCAGCTGAAG
CAGCAGCACCGGGACGCGGCGCAGCGGCTTAAGGAGCGGGACCTGCAGCTGCAGCAGCTGAGGGATGAGC
ACCAGCATCTGTTGGACTTGAGCAAGGACAGGAACTTGTTGGAACGCGAAAAGCTGCAGGCTCAGGTGGT
TGAACTGACCGCCAAAGTGCAGCAACAGGGTGAAACTATCAGCATGCTGCAGCGACGCATCGCGCTGGAA
GCCAAGAACTTCAGGCACCAGCTGCAGGCGGAGATCAATAAGCACAAAGACACGAGACACGACCTGGACC
TGGCCATCACTAACGCTGACAAACTATCCACTATTATCGAGATGAAGGAAAAGATGATAAGTACAGTCGC
AGGCAGAGCAGTGAAGTCTCCTACAAAGATTCCGTCTACCATCAACATTGCGAGACCGATTAGCAAGAGT
GGGAGAGAGGCTGCCAGAGGGACTGACGAGAGATCGAATATAATACAGTTAGAACAAAACCTACTCGCCA
AGCTATGTGAGAATTCACGGAATGTCAGTAGTTCACTATCGCACGAGGAAGACACAAGCTCATCCACAGA
ACCTCGCTCTAGATATGTCTCCTCTCGAAGTTCTACCAGCACCCGAACAACGCCGAGCCAAAACCAAAAC
CGAAAAGGATCAAAAGGTTCAGACGAAATAATAGAACTAGCAAAAACAGTACAAGAAGGCATGGCTGACC
TCACGATAGTTGACGACGATTTAAGAAATTCTACTCCTGAGGAAATGCAGAAAAGAATGGAAGCAATGAA
GGCTGATTTGTTAAATAAAATGAAGAATAACGAAGAACCCGGTTCTAGAAAGCCTAGTGCTTTGAGAAGA
AAATCTATGGAAGAGTCCATTGAGGAACACATTGAAGAAGTTGTGATAGAACGTCCGAAATCCAGAGGAA
GAAGAGGTTCTACGGTTTCGTTTTACGACAGTTCAAGTTCTGAAATGAACGCAGCAAGTAGCAGTACTGG
TGATGATGAGAAAACATTGTTAAAACGTGAGCTTAGCATAACTGATAAGAAAGCTACCGGGAAACCGATA
GACAAATATTGCAAAGACATCATTCAAGATATAGAGAAGAGCAGCAAAGTGATAGATAATCACATGAAAC
AGTTCACACAAACAAAATTCGCTAGTGATAAATTGATCGAGCAATTGCAAGCGGTTGACACGTTGAATGA
ATTTGTGAACGGTGGTGGAGACATACCACCAGAGGCATTCAGCGAAATGAACAATAATTTCAAAATGCTC
TCCGAACAAGTTTTTCTGAAAATGTTCCAGTGGCCCGTAAACGCAATCTTTCTGGAAGAAAAAACTCCC
GTATAGAATCTAGAACCAGTTTCCTCGGTGACTCCAATATGAGTAACCAGGATTTGCTCGAGGATTTGCT
GGGCAAGAAATGA

B3_P1(lebercilin)_1	GTCCCTGCCTCTATATCTTTCAACTGATTCTGCAGCTGCTTCACC
B3_P1(lebercilin)_2	GTCCCTGCCTCTATATCTTTGTTTCTTTTGGAGAGCACGTAACAC
B3_P1(lebercilin)_3	GTCCCTGCCTCTATATCTTTTCGCTGTGGGAGTTCAGCACTTGGG
B3_P1(lebercilin)_4	GTCCCTGCCTCTATATCTTTAAGCCGCTGCGCCGCGTCCCGGTGC
B3_P1(lebercilin)_5	GTCCCTGCCTCTATATCTTTTGTCCTTGCTCAAGTCCAACAGATG
B3_P1(lebercilin)_6	GTCCCTGCCTCTATATCTTTCCCTGTTGCTGCACTTTGGCGGTCA
B3_P2(lebercilin)_1	TTCTTGTAAATGATAGTGCGCATCATTCCACTCAACTTTAACCCG
B3_P2(lebercilin)_2	TCTCGTATCTCTGTAACGCGATTTCTTCCACTCAACTTTAACCCG
B3_P2(lebercilin)_3	TACCGGCTCTGCTGCACGCGCATCTTTCCACTCAACTTTAACCCG
B3_P2(lebercilin)_4	CTGCTGCAGCTGCAGGTCCCGCTCCTTCCACTCAACTTTAACCCG

B3_P2(lebercilin)_5 GCAGCTTTTCGCGTTCCAACAAGTTTTCCACTCAACTTTAACCCG

B3_P2(lebercilin)_6 CGTCGCTGCAGCATGCTGATAGTTTTCCACTCAACTTTAACCCG

lachesin

>lcl|XM_024078217.2_cds_XP_023933985.1_1 [gene=LOC112042983] [db_xref=GeneID:112042983] [protein=lachesin-like] [protein_id=XP_023933985.1] [location=418..1848] [gbkey=CDS]

ATGTCTGTTGACATTATGGCTGCGGAAGCGAGGAGGCGAGCGAGAGCCGTCGCCATGGAAACAGGGGGAC
TACCGCTGGTTTCTACGTTCGCACTTCTCACTATAGTTGCAGGCCAACTGCAGGGCTCGGGGTACGGCGA
CCCAGCCGAGCCGGAATTCCTGTCCCCATTGGAGAACATCACCGTCGCACAGGGGCGCGACGTTCATTTC
ACGTGCACCGTCAATCATCTCGGCACTTTTAAGGTGGCCTGGTTGAAGTCAGACACTAAGATGATATTGG
CGATGCACACACACATGGTGAACATCAACCCGCGTCTGTCCGTCACACACAACGGACACAACACTTGGAA
GCTGTATATCAGCAACGTACAGCCGAAGGACTCCGGCACTTACATGTGTCAGATCAACACCGACCCGATG
AAGAGTCAAATGGGACATCTGTCGGTCGTGATACCACCTGATATAGCAGACGATGATGGTTCCGAAGCCA
GTGCACCAGAGGGAGGGTCCGTGGAGCTTCGCTGTACAGCCACCGGAGTGCCGGAACCTACGGTATCGTG
GAAGAGAGCTGGCGGACGGAATATTATATTCCGCGATGAAGACGGCAGTGAATTGAAAGTGGTGGACAAC
TACGCAGGGACGACGTTGTCTCTGAGAGCGCTGAAGCGAGCGGATATGGGGACGTACCTCTGTATCGCTG
CGAACGGCATTCCTCCCACAAAGAGTCGACGCTACGAGGTGTCCGTACTCTTCGAGCCAATAGTAAGAGC
GGCAAGCATGGTCGTGTGGCGTGCAGCTGAGCTACAAGTCTCTCTGCAATGCTACGTGGAAGCCTCTCCC
AAGGCAATGACTATGTGGCAACGAGGGAAGTCACCAAATGGTGCAAAGCTATTAAATAGCTCAAAATACA
TAATATCCGAGAACTATTTGAACGAGTACGCGATGCGTATGAACCTGACAGTGAATCGGCTCAAGAAGAG
TGACTTTGGAGAGTACACTTGCTTAGCGGGCAATGCTTATGGGAAAGCGAATGTGACCATCACATTAAAA
GAAACGCCGCAGAAGACGACAACAACAACAACAACTACGACAACCACGACAACAACAACGGAACGCACAA
CTGCCGCAGCGACAACTCGGCCACCAAAACGACACCACAACAAGAAGCAAAAAGACAGAAACCAGAACAC
AGTCGACGTAGAAATTCACGAAATCAACAACGCACTCAATTTATACAACGTGAATGCATATGGACACTCG
AATAGTACGCAAAATGAATACGCCCAGAGGACAGAGAGGCAGAAAGTGAGGCCTTCTGGCCCGCCTCGAC
CCTACGTCGTTTACAACAACGCTACATTACAACAACGCTACATCACAACAATGCAATATTTATTTATTAT
CTTTGTCATAACTAGTTTATTAATATTGTAA

B1_P1 (lachesin)_1 GAGGAGGGCAGCAAACGGAAAATGGGGACAGGAATTCCGGCTCGG

B1_P1 (lachesin)_2 GAGGAGGGCAGCAAACGGAAAAAGTGCCGAGATGATTGACGGTG

B1_P1 (lachesin)_3 GAGGAGGGCAGCAAACGGAAGGTTGATGTTCACCATGTGTGTGTG

B1_P1 (lachesin)_4 GAGGAGGGCAGCAAACGGAATCCTTCGGCTGTACGTTGCTGATAT

B1_P1 (lachesin)_5 GAGGAGGGCAGCAAACGGAACACGACCGACAGATGTCCCATTTGA

B1_P1 (lachesin)_6 GAGGAGGGCAGCAAACGGAAGAAGCTCCACGGACCCTCCCTCTGG

B1_P2 (lachesin)_1 CGCCCCTGTGCGACGGTGATGTTCTTAGAAGAGTCTTCCTTTACG

B1_P2 (lachesin)_2 AGTGTCTGACTTCAACCAGGCCACCTAGAAGAGTCTTCCTTTACG

B1_P2 (lachesin)_3 GTCCGTTGTGTGTGACGGACAGACGTAGAAGAGTCTTCCTTTACG

B1_P2 (lachesin)_4 TTGATCTGACACATGTAAGTGCCGGTAGAAGAGTCTTCCTTTACG

B1_P2 (lachesin)_5 ATCATCGTCTGCTATATCAGGTGGTTAGAAGAGTCTTCCTTTACG

B1_P2 (lachesin)_6 GTTCCGGCACTCCGGTGGCTGTACATAGAAGAGTCTTCCTTTACG

Dscam2

>lcl|XM_024079361.2_cds_XP_023935129.2_1 [gene=LOC112043786] [db_xref=GeneID:112043786] [protein=cell adhesion molecule Dscam2-like] [protein_id=XP_023935129.2] [location=217..4722] [gbkey=CDS]

ATGAACATCGTCTGGATTATCGTGATAACATCTTTGTGGCATTCGGTTGTTGCTACAACTATAAACTTCA
TTGAGTCGAAGAAACACGAAGACCACAATCTTCTCTTCAAACAATATTACAGGGAGCCCGCATCCTTCCC
CGAGGCCGGGATTAGTGATAAGATCCCGGTTGACAAGCGGGATTATGGTACACAGTTTCAAAGTTTAGAA
TCAACGCAAAATGTAAGAAAGTCTAGGTCAAACGATCACAAGAAAACTAAAAGAAAATATGAAATTGAGC
AGCCTTTGCAGACCGTGACAATTGGTACAAGATTTAAAAGGGATCTAAGTTCAGGGAACGTTATGATCAC
GCAGCATTTCAACGATAAAGTTTTATCGCCGGGTGAAGACATTAGTCTTCAATGTACAGCGAGTTCGGAT
CGGCCACCGCGGTTCATTTGGGAGAGGGATGGTGTAGTCATCACGTCAAATACTGACTCTAGGTATATTT
TAGGACAAATGATGTCACCGACGGGGATCGGCGTAATATCACAATTAAACATATCCAGGTCAAGAGTTGA
AGACGGTGGACTCTACGCGTGTGTGGCACTCGAAGGGGAATCCACGGCTAAACACGCTGCTAGAATAGAC
GTCTATGGTCCACCGTACATAAGAACTTTGCCACCGATAAAAGTTCAAAGTGGTGATTCTTTAAAGCTTA
AATGCCCTTACTATGGATTTCCTATCAGTAAACTAGAATGGGAGCACAGAGGAAAGAAACTTATAAGCTC
ATTATTACCGCAACACACGAGGTATAAGCGAACCAATATAAGTAGTAAAAATCGTAAAGGCGCAAGAAAG
AGACGAAGAAAAAGGCAAGTCTTGGAAACTACGGAAGACGGAGTTCTTACAATAGAAAGAGTTTCAAAAG
AAGAAAATGGAGAAATGTACACATGTATAGTATTTAGTCCGTCAGGAGAAATGGCTAGAAGATCTTTTGA
AATACAAGTTGTAGAAGCGCCAGAATTGGACGAACTACGAGTAGGTTCGGGACTGAAGGAAGGACAGATT
GTACAAATTACTTGCAACATTATCAGTGGAGATCCACCAATTTTCTTTTCTTGGCTCAAAGATGGCATGA
AAATTCCTGCTAGCTTGAAAATAAACGAACGAAGTTCAGAACTATTTAGCGTTTTGATTATCAAAAGAGT
ATCGTTGGAGCATTGCGGTAGATACACGTGTATAGCTACAAATCATGTTGGTAAAGTTAATCAAACAACT
GACCTGTATATAAATGTTGCACCAAAGTGGGTGGAAGAACCAACGAACTCATCACTTCTTCTTGGTCAAC
GTGGCATTGTAGACTGTAATGCTAATGGATATCCCACGCCGCAAATACATTGGATGAAAAGGGATGCTGC
ATTAGGAATCTGGCGACCAATTCTCGATCTAGCTGGTGGTGGAGTTTCTAGTTATCCCAATGGTTCATTA
TCTTTAGAAGTGGTGTCTCTAGCAGATGAGGGAGAGTATGCGTGTCACGTAGATAATGGAGTCGGAGAGC
CATTGCACAAGAATTTATGGATAAGTGTCAATAAGCCGGTGCACTTCGAGTCCGTGGGAATGAACTTGAC
CACAAAGATGGGTCTACCGATGACTCTTATTTGTCAACCGTTGGGAGACAATCCTATACGGATTAAATGG
AGCTTAGACGGAAAACCCGTGGAATTTACATCCTCAAGAATCACAATATCAGAGTCGGTGAATTCAAATG
GTATGAAAAGCACAATCAATATCAACTATGTGGAAGGAAGAGACGGTGGAACTTACGAGTGTAGAGCGAG
CAATCCATACGGTGTTGCCACGCTTAATATTCATTTAAATATATTGGAACCACCCACGCCTCCAATGGAT
CTCCAAGTGGACTCTGTAACCAGTTCATCAGCGAAGTTGTCTTGGAGGGACACGATTGTATCCCACGTCC
AATACTACAGCCTACAGTACTCTGCCAACCAATACACGATGTGGGAATCAGCTAAAACAATTAATATAAC
CAGACAAGAAAGCGACATTCGACAAAACATAGAACTACGCGACCTGCAACCAGCGGTGGACTATCGAGTG
CGAGTTGCTTCCGGCAACCAAGTCGACCTCAGTCCGTACACTCAGCCTGTACACTTCACAACCTTACAAG
AAGCACCATCATCAAGCCCTCTTGGGGTGCAAGTTCAGCAGACAGACAACCCGGGCGAATTGCTCGTATC
ATGGATACAGCCATCTAGGGAGACCCACAACGGAGCATTACAGGGCTACCACGTGAAAGCGGTGCCAAGG
ATCAGTGGAGAAACTGGTTCAAATGATTCTCAAACTAAATTAGTAAAGGTTACATCAAGAAAAGGAAAAC
AAGAGACTATATTGAGTGGTCTCCTAAAGAACACGAGGTACGCAGTGTCTGTGAGTGCATTCAATTCAGC
GGGAAATGGTCCATTTTCTTTACCTGTTTATCAAACTACGAGAGAAGGTGCCCCAGAAGAAGCGCCATCT
AGTGTGGAATGTGTTGGATCTTCGTCTTCATCAGTGCGCGCAGCGTGGCGACCGCTAGCTAACTCTCACT
CATTGTTGGGATATGTTACACATTACTGCACGGACGATGGTCCCTGGCTAAACGTCACAACGCCGCATAC
AGAGTTGTATCTTCAAGGGCTGCTCAAATACACTAACTATACAATAAAAGTTGCCGGTTTCTCTAACTAT
GGCATCGGACCTTTCTCCTATCCTGTTGTGTGTACCACATTACAAGATGTTCCCGGACCACCAGCCGCTA
TAAAAGCGTTGATATCGTCTCCTACTTCTCTTCTTGTGAGTTGGAAACGACCTGATCAGCCTAATGGAGA
AATAACACATTATACTGTGTACGTCAAACCAGTTACCAGTACCAACGCCCCGCAAAGCTACAGGGTGGAA
CCGATACAGGAGTCGAACCTGTCCCGTCAGCTGACGTTCCCCCTCCCCGGGCTGAGCACGGGCATGCAGT
ACGAAGTGTTTGTGCGCGCGCACACCACTGCCGGGGAGGGCGCTCTAAGCAACAGGGTACACGTGGAACT
CACTTCCAGAGTAGTGGCAGGCGTAGCATCCCTCGGCGGGGCACTGTCGGTGGGGGTTGGGAACTCCCTG
CTGCTCGTGTGCCAGTGTGTGGGGTCGCCCCCGCCGCGCACCGTGTGGTACCACAAGCACAACATCATCA
CACACCACCCCAGGTTCACGAGGAACCACGATGACAGCTTACTGATTAATAATATAGACCAATCACTAAG
CGGGAACTACACTTGCTTGGCCAAGAACTTGTATGGCTCTGATTCAGTTTCGTATGAAGTGTCGGTTTTA
CCCACACCAGAACCCCCAGTTCTGAGGGTAACTTCACACAAGAATGCATTACATCTGCAATGGGACCACC
CGAGAAAAGCTGGAGAGAAAAGTCAGAAGATCATTTACGAGCTGACATGGAAAGAAGCGAACGGTTTGTG
GCAAGACACATGGTCGGACAAAGGAACCACTATTCAATCTGTACAGGAATACATATTAGAAGGTCTGAAA
TGTGGCACTAAATACTCTTTGAGGATGACAGCAGCCAATAGTGTTGGTTCCTCACAGCCAGCCTATGTAG
ATGCCACAACTTTAGGTGGAGTTCCCATATCACCAACAACAACGGAATGGTTTTGGAGTAACTCAAGTCA
TATCTACATACAACTGAGTGGTTGGGATGAGAATGGCTGTGAAATTACTCGATGGGAAGTTGACTACAGA
GAATACGGAGGAAAAGTTTGGAGAAGAGCGGAAAATCGACTGCCCCTACTTGACCAGTCGTGGGGTCATT
ACTCATCATCAATACTGAATCAACCGAACTCCTTCGTCATAGCCGACCTCGTGCCGGCGCAGTGGTACCA
AGTGAGGATAGTGGCTGAGAACGCGGCGGGCATATCTACGTCGCTTTATACGTATGCTACTACTACGATA
CTAGGAGAATCAATAGGTCCACCATCAGACAACTTCGACATAAACATGCTGGTCATCGTGTGCAGCTGTA
TACTGCTGTTGATTTGTTTAGTAACATGCATGTATATTCTAGTCAAAAAACATCATCACCAAAGACTAAC
AGAATACAGAAACTCACTGACAGGCGAGTGTAAGTCTGAAAGAAGCAACGCAACAGTCAACACACCTCAA
AGCATTCCCGCTGAAGTTAATAACAGAGTCTACAGTACGCCGGTGCACCTCACTTCAGATAATAAACATG
AATTATACGAAATAAGTCCGTACGCACAGTTCGCCATAGGTTTCCGAACGTTCGGTCATGTCGACAACCA

GGAGGTGCCCACTCGGATGCATTTGCCTGGCAACAGCAAAGCTAGATATGATAGTGAGACAAGTTTCCAA
ATGAGATCAGAATCCGAAGAAAGTGACTGCGTCTCACGAACTACGACATTGAAAAGTGCACCAAGAAAAG
CCTGTCGAGTGCCTCACCACAGATAA

B1_P1 (dscam2)_1	GAGGAGGGCAGCAAACGGAAACCGGGATCTTATCACTAATCCCGG
B1_P1 (dscam2)_2	GAGGAGGGCAGCAAACGGAATGACCTAGACTTTCTTACATTTTGC
B1_P1 (dscam2)_3	GAGGAGGGCAGCAAACGGAATTGTACCAATTGTCACGGTCTGCAA
B1_P1 (dscam2)_4	GAGGAGGGCAGCAAACGGAAGGCGATAAAACTTTATCGTTGAAAT
B1_P1 (dscam2)_5	GAGGAGGGCAGCAAACGGAAATCCCTCTCCCAAATGAACCGCGGT
B1_P1 (dscam2)_6	GAGGAGGGCAGCAAACGGAACGATCCCCGTCGGTGACATCATTTG
B1_P2 (dscam2)_1	AACTGTGTACCATAATCCCGCTTGTTAGAAGAGTCTTCCTTTACG
B1_P2 (dscam2)_2	TTTTCTTTTAGTTTTCTTGTGATCGTAGAAGAGTCTTCCTTTACG
B1_P2 (dscam2)_3	TCCCTGAACTTAGATCCCTTTTAAATAGAAGAGTCTTCCTTTACG
B1_P2 (dscam2)_4	GTACATTGAAGACTAATGTCTTCACTAGAAGAGTCTTCCTTTACG
B1_P2 (dscam2)_5	GTCAGTATTTGACGTGATGACTACATAGAAGAGTCTTCCTTTACG
B1_P2 (dscam2)_6	TGGATATGTTTAATTGTGATATTACTAGAAGAGTCTTCCTTTACG

homeobox protein araucan-like isoform X1

>lcl|XM_024082035.2_cds_XP_023937803.1_1 [gene=LOC112045732] [db_xref=GeneID:112045732] [protein=homeobox protein araucan isoform X2] [protein_id=XP_023937803.1] [location=231..1658] [gbkey=CDS]

ATGATCGTCCCTATGGCAGCGTATGCACAGTTCGGCTACTCCTACCCCTCCGCCTCGCAGTTGCTGGTGG
GCAGCAGCGGCGGCGGGAACGGGGGAGCGGCGACGTCGCCCGACGGCGGGTCCAGCTCCGGCCCGCCGCT
GTCGCCCGGCGGGTCGGGGGTCGCTGGCGGCGCACTGTCACCGGGGGCCGGCTCGCACGCCAGCACCCCC
GCCGCGCCGTGCTGCGACACCCCGCGCCCCATCATCACCGACCCCGTGTCCGGCCAGACGGTGTGCTCGT
GCCAGTACGACGCCCGGCTAGCGCTCTCATCGTACCCGCGCTTGTCCAGCGCCGCGGTCGGCGTCTACGG
AGCGCCGTACCCGTCCACGGATCAGAACCCTTACCCCAGCATCGGGGTTGACAGTTCGGCGTTCTACTCG
CCTCTGAGCAACCCGTACGCTCTGAAAGAGGGAAACGGCGAGATGTCAGCTTGGACATCGGCGGGCCTTC
AACCCTCCGGCGGCTACTACCCCTACGACCCCACGCTCGCGGCCTACGGATACGGAGCGGGGTACGATCT
AGCAGCCAGGCGGAAAAACGCGACGCGCGAGTCCACCGCAACACTAAAGGCGTGGCTCAACGAGCACAAG
AAGAACCCGTACCCGACCAAGGGGGAGAAGATAATGTTGGCCATCATCACCAAGATGACGCTGACGCAGG
TGTCCACGTGGTTCGCGAACGCGCGCCGGCGGCTCAAGAAGGAGAACAAGATGACCTGGGAGCCCAAGAA
CAAGACAGACGACGACGACGACACCATGCTGTCCGACGAGGAGAAAGACGACGACAAATTAAAACCAAAC
AAAGATGAGGAAAGGAAGGGGGACGAGCTTCTCCAGGGCATGCACAGTCAACTACTCGGCTACGGGATAA
AGGAGGAGTCCAAGCGAGGGACTTCAGACTGCGGCGTGCCGATACCAGCGTCGAAGCCCAAGATTTGGTC
TTTAGCAGACACCGCGGCATGTAAAACGCCGCCACCGGCCGCGCAGCCGTGGCCTCAGCATGGATACGGG
CCAGGGCCGGAGCGGTCGGTGTCTGACGGTGGTGCAAACGGGTTCGCACTACCTGCGACGGCGGCCGCGA
GTCCAGCGAGCGGGTCGTATGGGAGATACGGCGGTTTCCCCGGACAGTACAACCAGCATCCGTGCGTGCA
TCCAGCGGCGTTCCCCGACGTTCAGACTGACACTCCGCCACAAACTCCGCCCAACATGAAGGTGCCCAGC
GTGGCCAACCCGCTGGGCAGCGGCGGCGGCTCCGGCTATTGTTTCCCTCGGCACCAGCAGTCGCCACAGC
GGGACCCCTACCACAACCCTCACCATGCGAACAACCATCAACCCAACCAGCACCACAACGAGGGTTCGGC
CGCCTTTAAACCGTTCTACAAAAGGTAA

B1_P1 (araucan)_1	GAGGAGGGCAGCAAACGGAACCGCCAGCGACCCCCGACCCGCCGG
B1_P1 (araucan)_2	GAGGAGGGCAGCAAACGGAAGGGGCGCGGGGTGTCGCAGCACGGC

B1_P1 (araucan)_3	GAGGAGGGCAGCAAACGGAAATGAGAGCGCTAGCCGGGCGTCGTA
B1_P1 (araucan)_4	GAGGAGGGCAGCAAACGGAAGGGTTCTGATCCGTGGACGGGTACG
B1_P1 (araucan)_5	GAGGAGGGCAGCAAACGGAACTCTTTCAGAGCGTACGGGTTGCTC
B1_P1 (araucan)_6	GAGGAGGGCAGCAAACGGAAGGTCGTAGGGGTAGTAGCCGCCGGA
B1_P2 (araucan)_1	TGCGAGCCGGCCCCCGGTGACAGTGTAGAAGAGTCTTCCTTTACG
B1_P2 (araucan)_2	CTGGCCGGACACGGGGTCGGTGATGTAGAAGAGTCTTCCTTTACG
B1_P2 (araucan)_3	CCGCGGCGCTGGACAAGCGCGGGTATAGAAGAGTCTTCCTTTACG
B1_P2 (araucan)_4	GAACTGTCAACCCCGATGCTGGGGTTAGAAGAGTCTTCCTTTACG
B1_P2 (araucan)_5	TGTCCAAGCTGACATCTCGCCGTTTTAGAAGAGTCTTCCTTTACG
B1_P2 (araucan)_6	CTCCGTATCCGTAGGCCGCGAGCGTTAGAAGAGTCTTCCTTTACG

ivory

ATTCGTCGTAGAGACCGCGAGCGGCGCGCGCGCGTTTCAACGAACTACGATTAAAAAAACA
GTTCATTCAGCCGCCTCGGGAGCAACGACCTCGCGGCCATTATTTTTCAACGAACGTCCTTGTCG
CGAACAGCTGATTCACGAATCCGTGCTCCGAATGCAAAGTGATTCCTTGACAGAATGGAAAATAA
GGTTTTTTAATCTCGTAGGTAATTTTCGATTGATACATTTTACAGTATAAAAATGTAGTGAGTCTAT
GTAGAACTATTTATTCTATATAACAATAAGGAATAGAGGAGTAACTGAAATTTGCAAAATTTGTTA
ATTATGACGTATTAGCGCTGAAATTTGTAAGCCCAATTTTAGTGTAGTGTAGTGTGGTAATTTTTCA
ATTTCAAAATGTGAAGTGTTTGTGTAAGTGATCGACATTGATCGGCAATCATCCAG

B3_P1(ivory)_1	GTCCCTGCCTCTATATCTTTgcgcgcggcgagcgGGTCTCTACGA
B3_P1(ivory)_2	GTCCCTGCCTCTATATCTTTGGTCGTTGCTCCCGAGGCGGCTGAA
B3_P1(ivory)_3	GTCCCTGCCTCTATATCTTTATTCGTGAATCAGCTGTTCGCGACA
B3_P1(ivory)_4	GTCCCTGCCTCTATATCTTTGAaattttttggaataaaaggtTTC
B3_P1(ivory)_5	GTCCCTGCCTCTATATCTTTAATAGTTCTACATAGACTCACTACA
B3_P1(ivory)_6	GTCCCTGCCTCTATATCTTTgtattaattgtttaaaacgAAATTT
B3_P1(ivory)_7	GTCCCTGCCTCTATATCTTTctttaactttttaatggtgtgatgt
B3_P2(ivory)_1	TTTTTAATCGTAGTTCGTTGAAtgcTTCCACTCAACTTTAACCCG
B3_P2(ivory)_2	GTTCGTTGAAAAAATAATGGCCGCTTCCACTCAACTTTAACCCG
B3_P2(ivory)_3	AGGAATCACTTTGCATTCGGAGCACTTCCACTCAACTTTAACCCG
B3_P2(ivory)_4	AATGTATCAATCGAAAATTACCTACTTCCACTCAACTTTAACCCG
B3_P2(ivory)_5	CTCTATTCCTTATTGTTATATAGAATTCCACTCAACTTTAACCCG
B3_P2(ivory)_6	GGCTTACAAATTTCAGCGCTAATACTTCCACTCAACTTTAACCCG
B3_P2(ivory)_7	ATCACTTACACAAACACTTCtgtaaTTCCACTCAACTTTAACCCG

Supplementary References

1. Bier, E. Drawing lines in the Drosophila wing: Initiation of wing vein development. *Curr. Opin. Genet. Dev.* **10**, 393–398 (2000).
2. De Celis, J. F. Pattern formation in the Drosophila wing: The development of the veins. *BioEssays* **25**, 443–451 (2003).
3. Blair, S. S. Wing vein patterning in Drosophila and the analysis of intercellular signaling. *Annu. Rev. Cell Dev. Biol.* **23**, 293–319 (2007).
4. Campbell, G. & Tomlinson, A. Transducing the Dpp Morphogen Gradient in the Wing of Drosophila. *Cell* **96**, 553–562 (1999).
5. Martín, M., Ostalé, C. M. & De Celis, J. F. Patterning of the drosophila L2 vein is driven by regulatory interactions between region-specific transcription factors expressed in response to Dpp signalling. *Dev.* **144**, 3168–3176 (2017).
6. Lunde, K., Biehs, B., Nauber, U. & Bier, E. The knirps and knirps-related genes organize development of the second wing vein in Drosophila. *Development* **125**, 4145–4154 (1998).
7. Cook, O., Biehs, B. & Bier, E. brinker and optomotor-blind act coordinately to initiate development of the L5 wing vein primordium in Drosophila. *Development* **131**, 2113–24 (2004).
8. Winter, S. E. & Campbell, G. Repression of Dpp targets in the Drosophila wing by Brinker. *Development* **131**, 6071–6081 (2004).
9. Minami, M., Kinoshita, N., Kamoshida, Y., Tanimoto, H. & Tabata, T. brinker is a target of Dpp in Drosophila that negatively regulates Dpp- dependent genes. *Nature* **398**, 242–246 (1999).
10. Tian, S., Lee, B., Banerjee, T. Das, Murugesan, S. N. & Monteiro, A. A novel Hox gene promoter fuels the evolution of adaptive phenotypic plasticity. *Nat. Ecol. Evol.* (2025) doi:10.1038/s41559-025-02891-5.
11. Tian, S. *et al.* A microRNA is the effector gene of a classic evolutionary hotspot locus. *Science (80-.).* **1141**, 1135–1141 (2024).
